# The influence of CpG and UpA dinucleotide frequencies on RNA virus replication and characterization of the innate cellular pathways underlying virus attenuation and enhanced replication

**DOI:** 10.1093/nar/gku075

**Published:** 2014-01-25

**Authors:** Nicky J. Atkinson, Jeroen Witteveldt, David J. Evans, Peter Simmonds

**Affiliations:** ^1^Infection and Immunity Division, Roslin Institute, University of Edinburgh, Easter Bush, Edinburgh EH25 9RG, UK and ^2^School of Life Sciences, University of Warwick, Coventry CV4 7AL, UK

## Abstract

Most RNA viruses infecting mammals and other vertebrates show profound suppression of CpG and UpA dinucleotide frequencies. To investigate this functionally, mutants of the picornavirus, echovirus 7 (E7), were constructed with altered CpG and UpA compositions in two 1.1–1.3 Kbase regions. Those with increased frequencies of CpG and UpA showed impaired replication kinetics and higher RNA/infectivity ratios compared with wild-type virus. Remarkably, mutants with CpGs and UpAs removed showed enhanced replication, larger plaques and rapidly outcompeted wild-type virus on co-infections. Luciferase-expressing E7 sub-genomic replicons with CpGs and UpAs removed from the reporter gene showed 100-fold greater luminescence. E7 and mutants were equivalently sensitive to exogenously added interferon-β, showed no evidence for differential recognition by ADAR1 or pattern recognition receptors RIG-I, MDA5 or PKR. However, kinase inhibitors roscovitine and C16 partially or entirely reversed the attenuated phenotype of high CpG and UpA mutants, potentially through inhibition of currently uncharacterized pattern recognition receptors that respond to RNA composition. Generating viruses with enhanced replication kinetics has applications in vaccine production and reporter gene construction. More fundamentally, the findings introduce a new evolutionary paradigm where dinucleotide composition of viral genomes is subjected to selection pressures independently of coding capacity and profoundly influences host–pathogen interactions.

## INTRODUCTION

Studies of RNA viruses provide major insights and functional understanding of replication mechanisms and host cellular interactions in which processes of mutation, fitness selection, recombination and sequence drift can be directly observed. The small size and necessarily compact arrangement of protein-coding sequences and replication elements creates a range of constraints on sequence change that are frequently both quantitatively and qualitatively different from evolutionary selection pressures on their eukaryotic and prokaryotic hosts. Coding regions of RNA virus genomes frequently contain secondary and higher order RNA structures that function as points of interaction with viral and host cellular proteins and RNA elements such as ribosomes (1,2). For example, *cis-*replicating elements of enteroviruses in the 2C-coding region and of hepatitis C virus in NS5B are revealed by a marked suppression of sequence variability at largely unconstrained synonymous coding sites (3,4). Substitutions in base-paired nucleotides in the stem loop are selected against if they damage the stability of pairing, and changes are often accompanied by compensatory changes in the paired residue to restore pairing. Many RNA viruses have also evolved additional genes to inhibit or divert innate cell defences, typically embedded within other viral genes in alternative reading frames (5), such as the PB1-F2 protein of influenza A virus (6). These interactions place further constraints on virus sequence change in coding regions of RNA viruses over and above their protein-coding function.

While these additional RNA-structure- or protein-coding-based constraints are relatively well understood in viruses where replication mechanisms are characterized, RNA virus genomes additionally evolve under a series of additional compositional constraints for which functional explanations are currently lacking. RNA viral genomes show an extremely wide range of base compositions; among mammalian viruses, G + C contents range from as low as 33% (respiratory syncytial virus) to 69.9% (rubella virus). Coding regions of RNA viruses frequently show highly abnormal codon usage patterns (7); hepatitis A virus show highly restricted codon usage [formally quantified by measures such as an effective number of codons [Enc] of 39.8; (8)] and codon choices different from mammalian usage [measured as a codon adaptation index of 0.76; (9)].

However, one of the most unexpected compositional abnormalities, particularly among RNA and small DNA viruses infecting mammals and plants, is the markedly biased frequencies of certain dinucleotides such as CpG and UpA throughout their genomes (10–12). For example, poliovirus shows a frequency of CpG dinucleotides in its coding region that is only 54% of the expected value calculated from its (mononucleotide) composition (based on a 23.5% [frequency of G] × 23.4% [frequency of C]). UpA is similarly suppressed (74% of expected value) while other dinucleotides are over-represented (CpA: 1.32×; UpG: 1.35×). The lack of suppression of GpC and ApU in RNA viruses (and host genomes) demonstrates that this effect is specific to CpG and UpA and does not simply reflect evolutionary pressure for regions of greater or lesser duplex stability.

The overlap between dinucleotides creates inter-dependencies that complicate analysis of the mutational and selection pressures that underlie these biased compositional frequencies. For example, the methylation-associated elimination of CpG dinucleotides through C->T transitions in genomic dsDNA creates TpG and ApC dinucleotides, a process that potentially accounts for their over-representation in vertebrate DNA (10) and indirectly depletes TpA (13). We recently developed a parameterized Markov method to identify what further dinucleotide context-dependent mutations best account for compositional patterns in genomic and cytoplasmically expressed mRNA sequences of mammals and a range of other eukaryotic phyla. This was extended to analyse compositional abnormalities of RNA viruses infecting mammals and insects (10). As expected, 11- to 14-fold greater frequencies of C→T transitions upstream of G (depicted C→T,G) than other transitions best modelled dinucleotide frequencies in mammalian genomic DNA. Additionally, further mutations that eliminated UpA dinucleotides and CpG dinucleotides were observed in the subset of sequences expressed as mRNA and entering the cytoplasm. Modelling provided evidence for equivalent selection against these dinucleotides in mammalian and plant RNA viruses that is consistent with their under-representation in previous compositional analyses (10,11,14). The nature of this apparent selection against CpG dinucleotides operating on both host and viral cytoplasmic RNA sequences remains unexplained functionally although it has been frequently hypothesized that recognition of CpG and potentially UpA motifs is part of an as yet uncharacterized self–non-self recognition system coupled to innate immunity (15). This may be functionally and perhaps evolutionarily related to Toll-like receptor 9 that recognizes non-methylated CpG dinucleotides in DNA sequences (14,16).

Further evidence that the presence of CpG dinucleotides in viral sequences either activate or are targets of cell defence mechanisms is provided by the observation that polioviruses with artificially elevated CpG frequencies in their genomic RNA were markedly attenuated and replicated to titres several orders of magnitude lower than wild-type virus *in vitro* (17). The existence of such recognition systems may in turn have placed additional selection pressures on host expressed mRNA sequences to evade these viral countermeasures. In the current study, we have developed a model system based on an infectious clone of the enterovirus, echovirus 7 (E7), to investigate the effect of modifying dinucleotide frequencies on virus replication in mammalian cells. The observation that elevated frequencies of both UpA and CpG attenuate viral replication while lowering frequencies below those of native viral sequences enhances replication provided the opportunity to investigate the role of different components of the innate immune system in dinucleotide recognition and recruitment of antiviral defences.

## MATERIALS AND METHODS

### Cell culture and cell lines

RNA transcripts of the pT7:E7 infectious cDNA clone of the isolate Wallace (accession number AF465516) and pRiboE7luc replicon were used to generate E7 viral stocks and the E7 replicon used in the study. Both were propagated in rhabdomyosarcoma (RD) cells using Dulbecco modified Eagle medium (DMEM) with 10% fetal calf serum (FCS), penicillin (100 U/ml) and streptomycin (100 µg/ml). All cells were maintained at 37°C with 5% CO_2_. Monolayer cultures of A549 cells and shRNA cell derivatives were used for the interferon pathway analyses while RD cells were used in all other experiments.

### *In silico* design of CpG- and UpA-modified viruses

Two regions of the full-length E7 cDNA pT7:E7 clone were selected for mutagenesis. These lay in regions of the genome bounded by the unique restriction sites SalI (genome position 1878) and HpaI (genome position 3119) for Region 1 and EcoRI (genome position 5403) and BglII (genome position 6462) for Region 2. To generate CpG-zero mutants (designated lowercase ‘c’), all CpG dinucleotides were eliminated by replacement of either the C or the G base with a randomly alternative selected base selected to preserve coding of the underlying sequence. A similar strategy was used to generate UpA-low (designated ‘u’) and combined zero CpG and low UpA mutants (‘cu’), with the restriction that UpAp(C or U) codons encoding tyrosine precluded elimination of all UpA dinucleotides. Introduction of as many as possible CpG or UpA dinucleotides while preserving coding was used to generate CpG-high and UpA-high sequences (uppercase ‘C’ and ‘U’, respectively). Sequence changes and their effects on base compositions of the resulting insert sequences are shown in Table 1. Sequences generated for the study are provided in Supplementary Data.

**Table 1.** Composition of e7 and luciferase insert sequences

Region	Sequence	Symbol	G + C content	Total changes	freq^a^	CpG total (change^b^)	O/E ratio^c^	freq	UpA total (change)	O/E ratio^d^	Codon usage		
											CAI	Enc	CP bias
**1**	Native	W	47.6%	–	0.041	51 (−)	0.730	0.050	62 (−)	0.742	0.685	56.5	−0.047
	Permuted	P	47.6%	142	0.041	51 (0)	0.730	0.050	62 (0)	0.742	0.694	55.8	−0.028
	CpG-zero	c	44.3%	56	0	0 (−51)	0	0.057	70 (+8)	0.741	0.693	47.3	0.042
	UpA-low	u	50.9%	43	0.045	56 (+5)	0.730	0.015	19 (-43)	0.256	0.672	51.9	−0.003
	Both-low	cu	47.5%	102	0	0 (−51)	0	0.015	19 (−43)	0.227	0.686	43.6	0.083
	CpG-high	C	56.5%	150	0.146	180 (+129)	1.828	0.042	52 (−10)	0.900	0.666	44.3	−0.222
	UpA-high	U	40.9%	119	0.032	38 (−12)	0.756	0.139	171 (+109)	1.593	0.726	45.8	−0.094
2	Native	W	47.1%	–	0.018	18 (−)	0.320	0.047	48 (−)	0.695	0.743	52.9	0.023
	Permuted	P	47.6%	109	0.018	18 (0)	0.320	0.047	48 (0)	0.695	0.739	48.8	0.017
	CpG-zero	c	45.5%	21	0	0 (−18)	0	0.047	48 (0)	0.654	0.739	50.1	0.085
	UpA-low	u	50.0%	34	0.021	21 (+3)	0.331	0.014	14 (−34)	0.215	0.781	48.7	0.050
	Both-low	cu	48.5%	55	0	0 (−18)	0	0.037	38 (−10)	0.824	0.788	47.1	0.121
	CpG-high	C	56.4%	124	0.133	135 (+116)	1.667	0.037	38 (−10)	0.824	0.658	40.3	−0.247
	UpA-high	U	39.2%	107	0.015	15 (−3)	0.390	0.149	151 (+103)	1.633	0.606	39.0	−0.048
Luc	Native	L	45.3%	–	0.063	103 (−)	1.242	0.052	85 (−)	0.699	0.647	45.0	−0.103
	Both-low	I	43.2%	176	0.006	1 (−102)	0.013	0.018	19 (−66)	0.145	0.740	0.74	0.118

^a^Frequency of dinucleotide in insert region.

^b^Total number of CpG and UpA dinucleotides in sequence. Changes in numbers between mutated and original WT sequence are indicated in parentheses.

^c^Ratio of observed dinucleotide frequency (O) to that expected based on mononucleotide composition (E) i.e. f(CpG)/f(C) * f(G).

^d^O/E ratio based on f(UpA)/f(U) * f(A).

The codon adaptation index for human codon usage was calculated through the website http://genomes.urv.es/CAIcal/(9). The Enc (8) and codon pair bias [CPB; (18,19)] were calculated using the program Composition Scan in the SSE package (20). The CPB value was the mean of codon pair scores listed in Supplementary Table S1 (19) corresponding to each codon pair in the insert sequences.

### RNA structure prediction and sequence variability

Prototype sequences of each species B enterovirus (http://www.picornaviridae.com/) were scanned for RNA secondary structure using the program Folding Energy Scan in the SSE package using 200 base fragments incrementing by 152 bases and 50 sequence-order-randomized control using the algorithm NDR that preserves dinucleotide frequencies of the native sequence (21). Mean folding energy difference (MFED) values for each fragment were plotted against the mid-point of each fragment to localize areas of sequence-order-dependent RNA secondary structure. MFEDs were also similarly calculated for the reverse complement of each genome sequence. Synonymous sequence variability was determined by measurement of mean pairwise distances using the program Sequence Scan in the SSE package.

### Clone construction and recovery of mutant viruses

The full-length E7 cDNA pT7:E7 clone under the control of a T7 promoter was used for this study. Mutant E7 constructs with altered CpG/UpA content were generated by ordering custom DNA sequences (GeneArt, Life Technologies, Paisley, UK). Sequences were provided in standard antibiotic-resistant cloning vectors and were cloned into pT7:E7. All clones were sequenced over the insert regions prior to further applications. To recover the mutant viruses with altered CpG/UpA content, assembled plasmids were linearized using NotI and RNA generated using a Mega Script T7 *in vitro* transcription kit (Ambion). 100 ng of RNA was transfected into RD cells using Lipofectamine 2000 (Invitrogen) according to the manufacturer’s instructions. The resulting cell lysates were used to generate passage 1 stocks by re-infecting RD cells. Viral titres were determined by TCID_50_ titration in RD cells.

### Replication phenotype

RD cells were seeded at 5 × 10^5^ cells per well in six-well plates and subsequently infected with the wild-type (WT) or CpG/UpA mutants at an multiplicity of infection (MOI) of 0.01 per cell for 1 h before removing the inoculum and washing the cells. Samples were then withdrawn at given time points (12, 18, 24, 30 and 42 h post-infection) and the viral titre determined by measurement of TCID_50_s in 96-well format plates. The assay was performed in triplicate for each virus. For plaque assays, confluent RD cells in 100-mm dishes or 6-well plates were inoculated with virus in DMEM and incubated for 1 h at 37°C with occasional rocking. The inoculum was removed and replaced with an overlay consisting of 2% Methocell (MC, Sigma) in DMEM. Plates were incubated for 96 h at 37°C, fixed with 3.5% formaldehyde and stained with 0.1% crystal violet. Plaque sizes were quantified using ImageJ software.

### Quantitative real-time polymerase chain reaction

RNA was isolated from cells using the RNAspin Mini Kit (GE Healthcare) or from viral supernatant using the QIAamp Viral RNA Mini Kit (Qiagen). Reverse transcription was performed using M-MLV reverse transcriptase (Promega) and random primers The qPCR reactions were done using Sensifast SYBR Hi-Rox master mix (Bioline) in a Rotorgene-Q cycler (Qiagen) using the primers listed in Supplementary Table S1. For E7, a standard curve using quantified PCR product was carried out in parallel, allowing quantification of viral copy number. RNA to infectivity ratio was determined by viral load measurements of RNA extracted from 5000 TCID_50_ units of each virus.

### siRNA knockdown of PKR

Double stranded RNA-dependent protein kinase (PKR)-specific siRNA (EIF2AK2, Ambion Silencer Select validated siRNA) was used at a concentration of 33 nM. Transfections were performed using Lipofectamine 2000 (Invitrogen) using 1 μl of lipofectamine per 24 well according to the manufacturer’s instructions. Cells were incubated for 48 h before further experiments were performed. Validated non-targeted control siRNA was used at the same concentration and incubation period. To quantify the degree of knockdown of PKR by siRNA, equal amounts of protein extracted from cell lysates 48 h after siRNA transfection were blotted and detected using a PKR-specific mAB (Ye350, Abcam) followed by anti-rabbit HRP-conjugated antibodies (SA1-200, Pierce antibodies) and ECL detection. Band densities were measured for two independent experiments. PKR mRNA expression was quantified at the same time point by qPCR using PKR-specific primers.

### Replicon construction and replication kinetics

To accurately quantify intracellular viral replication, the pRiboE7luc sub-genomic replicon plasmid was used. This contains a version of the E7 genome in which the structural genes (nucleotides 753–3118) are replaced with the 1704-bp-long firefly luciferase gene. A synthetic version of the luciferase gene with its CpG and UpA dinucleotides removed while maintaining its coding sequence was designed, which also included a CpG- and UpA-low 72-bp linker sequence at the 3’ containing a SanDI restriction site. The sequence was cloned into pT7:E7 using the unique restriction sites KasI (genome position 781) and SanDI (position 3191). To create replicons containing the additional Region 2 CpG or UpA low inserts (designated ‘l|-|c’ and ‘l|-|u’, with the original clone designated as ‘L|-|W’), a 3235-bp section of the replicon directly downstream of the luciferase gene was excised using SanDI and BglII restriction enzymes. This was then replaced with the equivalent sections of the previously described c|c or u|u constructs with their modified Region 2 sequences. Replicon plasmids were linearized using NotI; RNA were synthesized *in vitro* using T7 RNA polymerase (Ambion); and RNA integrity was confirmed using an 2100 Bioanalyser (Agilent) before use.

Assays were performed by transfecting 50 ng of replicon RNA into RD cells seeded at 3 × 10^4^ cells per well in 96-well plates. RNA was transfected at given time points (1, 4, 6, 8 and 12 h) before luciferase assays were carried out using the Luciferase Assay System (Promega), according to the manufacturer’s instructions. Cells were lysed using the Passive Lysis Buffer, and the cell lysate was transferred to opaque 96-well plates for luminescence analysis using the Glomax Multi Detection System (Promega).

### Sequencing of individual virus genomes

Viral RNA was isolated from E7 WT, R1/R2 CpG-high or R1/R2 UpA-high virus stocks generated in RD cells, and cDNA created. Nested primers were designed to amplify a ∼500-bp section of the modified Region 1 (nucleotides 1835–2363) and an unmodified region of E7 (nucleotides 3241–3723) using primers listed in Supplementary Table S1 (Supplementary Data). The proofreading enzyme *Pfu*Turbo DNA Polymerase (Agilent) was used to amplify the two sections from each cDNA. The products were purified, cloned into a TA vector (pGEM-T easy, Promega) and transformed into competent *E. coli*, generating a separate colony for each copy of the original viral cDNA. The 500-bp inserts were sequenced using M13 primers.

### Competition assays

Equal titres of WT and mutant virus (combined MOI = 0.01) were applied simultaneously to RD cells in 24-well plates. Following CPE, the supernatant was frozen, thawed and 200 µl applied to fresh RD cells. This was continued for up to 10 passages, and performed in triplicate for each virus competition. For the pairwise competition assay, RD cells were inoculated with paired combinations of seven viruses, giving 21 combinations in total. Each pairwise assay was carried out in a single well and passaged through RD cells 10 times. RNA was isolated from the final supernatants; cDNA was generated and nested PCR carried out to amplify either Region 1 or Region 2 using primers listed in Supplementary Table S1. The amplified fragment was then subjected to restriction endonuclease cleavage to estimate the proportion of each sequence (enzymes listed in Supplementary Table S2).

### Early intra-cellular replication kinetics

For synchronized infections, RD cells were infected at 4°C with a total of 2 × 10^8^ genome copies (1000 per cell) and maintained at 4°C for 30 min before being moved to 37°C. Cells were washed twice with PBS and then trypsinized 1 h or 4 h post infection. The cells were then pelleted and washed again in PBS before RNA was isolated and viral copy number determined by qRT-PCR. Copy number was normalized against the housekeeping gene GAPDH by qRT-PCR primers (primers listed in Supplementary Table S1).

### Replication of virus with exogenous interferon-β

RD cells in 24-well plates were pre-treated before infection with 1000 U/ml human interferon-β (IFNβ) for 24 h, or mock treated with DMEM. Cells were then infected with WT or mutant E7 at an MOI of 1 for 1 h before the inoculum was removed and replaced with media containing the same concentration of IFNβ as previously. Eight hours post-infection, RNA was isolated as described above. Viral copy number was determined by qRT-PCR as described previously.

### Analysis of viral growth following inhibition/knockdown of signalling pathway components

RD cells in 24-well plates were treated 45 min before infection with 2-aminopurine (2-AP, dissolved in PBS–glacial acetic acid 200:1) and C16 (dissolved in DMSO) at final concentrations of 5 and 2 µM, respectively. RD cells were treated 2 h before infection with the cyclin-dependent kinase (cdk) inhibitor, roscovitine (dissolved in DMSO), at a final concentration of 40 μM. All inhibitor experiments used mock-treated cells with their respective solvents alone.

Cells were infected with WT or mutant variants of E7 at MOIs of 0.03–0.1, and viral RNA was isolated from cells 24 h later. Viral load was quantified using qRT-PCR. Cells expressing PKR, retinoic acid-inducible gene-I (RIG-I) or melanoma differentiation associated gene 5 (MDA-5) shRNA were used to investigate the effect of PKR, RIG-I or MDA5 knockdown on viral growth. shPKR, shRIG-I and shMDA5 cell lines, as well as the parental A549 line, were infected with WT or mutant variants of E7 at an equal RNA copy number relating to a wild-type MOI of 1. Viral RNA was isolated and quantified 8 h post infection.

### Innate and adaptive immune responses PCR array

RD cells in 24-well plates were infected with E7 WT or mutant viruses at an MOI of 10, or a consistent viral genome copy number equating to an MOI of 10 in the WT. Sendai virus and Poly I:C, strong inducers of IFNβ, were separately inoculated as positive controls. Viral RNA titres were confirmed in virus stock preps using qRT-PCR. Cells were harvested 8 h post infection, and RNA was isolated. RNA from six biological replicates was combined, and RNA integrity number was confirmed to be greater than 9.7 for all samples using a 2100 Bioanalyzer (Agilent). cDNA was made using the RT^2^ First Strand cDNA Synthesis kit (Qiagen) with 800 ng RNA per sample. The relative expression of 84 candidate genes was then analysed with pre-made ‘Human Innate & Adaptive Immune Responses PCR Array’ RT^2^ Profiler PCR Arrays (SABiosciences, catalogue number PAHS-052), and RT^2^ SYBR Green Rox Fast Master Mix (Qiagen). Cycling conditions were 95°C for 10 min, followed by 40 cycles of 95°C for 15 s and 60°C for 30 s. Data were normalized and fold changes calculated using the PCR Array Data Analysis Web Portal (SABiosciences).

## RESULTS

### Strategy for maximizing or minimizing CpG/UpA content in mutant viruses

Like other small RNA viruses, the frequency of CpG dinucleotides in the E7 genome is suppressed relative to the expected frequency based on its G + C content, with an observed to expected ratio of CpG dinucleotides in the coding sequence of E7 of 0.58. Frequencies of UpA dinucleotides are also suppressed in the E7 genome (observed to expected ratio of 0.78).

To investigate whether CpG and UpA dinucleotide frequencies influenced the ability of E7 to replicate *in vitro*, we created a series of mutated viruses in which frequencies of both nucleotides were changed from their native levels. This was achieved by reverse genetics using the pT7:E7 infectious clone. RNA transcripts generated from a linearized plasmid containing the E7 complete genome sequence generate infectious virus for phenotypic characterization after transfection into a wide range of mammalian cells.

**Figure 1.**
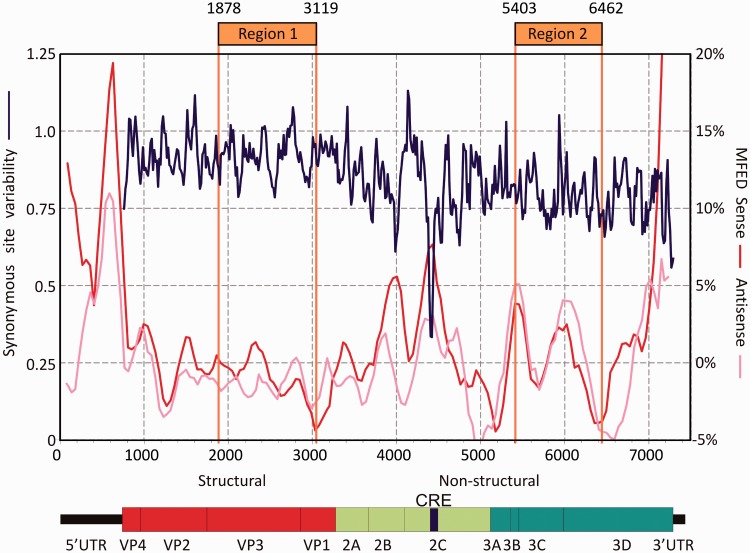
Genome organization of E7 and positions of mutated insert regions. Insert positions are compared with genome diagram and a plot of sequence variability within species B enteroviruses at synonymous sites (blue line) and folding energies indicative of RNA secondary structure (red and pink lines). Variability at synonymous sites (left y-axis) was computed at each codon position in alignments, plotted with a window size of 41 codons. MFED values (right y-axis) for sense and antisense RNA sequences were calculated for 200 base fragments, incrementing by 48 bases; values plotted represent mean values of five consecutive fragments. Nucleotide positions were calculated relative to the pT7:E7 cDNA sequence.

To select areas for mutagenesis, we sought to avoid regions of the genome that contained RNA elements required for replication or translation functions of the virus, such as the *cis*-replicating element (CRE) embedded in the 2C coding sequence (2). By scanning an alignment of complete genome sequences of each of the currently described species B serotypes (including the pT7:E7 sequence of the infectious clone), an area of marked suppression of sequence variability co-localized in the 2C region with the CRE (Figure 1). Calculation of folding energies to detected RNA secondary structure in the genome showed prominent regions of structure in the 5’UTR, 3’UTR and the CRE. MFED values were consistently higher for species B sequence orientated in the plus (genomic RNA) orientation. The remainder of the genome showed no evidence for consistent RNA structure formation (MFED values around zero) (Figure 1).

The combination of unrestricted synonymous variability and an absence of RNA secondary structure over long stretches of the E7 genome provided opportunities for altering dinucleotide frequencies without disrupting potential replication elements embedded within the coding sequence. Two genome regions (occupying positions 1878–3119 and 5403–6462, individually corresponding to 16.7% and 14.2% of the full length genome) were selected for mutagenesis based on these criteria. Sequences were modified by replacing nucleotides within CpG or UpA dinucleotides with alternative bases that preserved coding. It was possible to remove all CpG dinucleotides from both regions and reduce UpA to frequencies approximately one third of wild-type levels (Table 1; CpG-zero and UpA-low insert sequences). As an alternative strategy, to maximize frequencies of these dinucleotides, every site that could tolerate the creation of these dinucleotides without changing coding was identified and mutated to create sequences with 2.5–3x their naturally occurring frequencies (Table 1; CpG-high, UpA-high). To ensure that sequence disruption did not damage or destroy undetected replication element(s) within Region 1 and 2, sequences were permuted using the algorithm CDLR in the SSE sequence package (E7-permuted in Table 1). This randomizes the order of codons within the sequence while maintaining coding and dinucleotide frequencies through swaps between equivalently coding triplets in the same upstream and downstream dinucleotide contexts. All mutated sequences were then synthesized and cloned into the pT7:E7 infectious clone using naturally occurring restriction sites.

### Replicative fitness of mutants with modified CpG/UpA frequencies

Wild-type E7 and mutant viruses were recovered in cell culture by transfecting whole-genome RNA sequences obtained through T7 transcription of pT7:E7. Recovered virus was then titred by measurement of TCID_50_ values and used in subsequent experiments. In the following sections and associated figures and tables, wild-type and permuted control sequences were designated as W and P; high CpG and UpA mutants as U and C and low mutants as u and c. Clones in which region 1 was exchanged were designated as U|W and C|W for UpA and CpG-high sequences respectively; region 2 as W|U and W|C etc.

**Figure 2.**
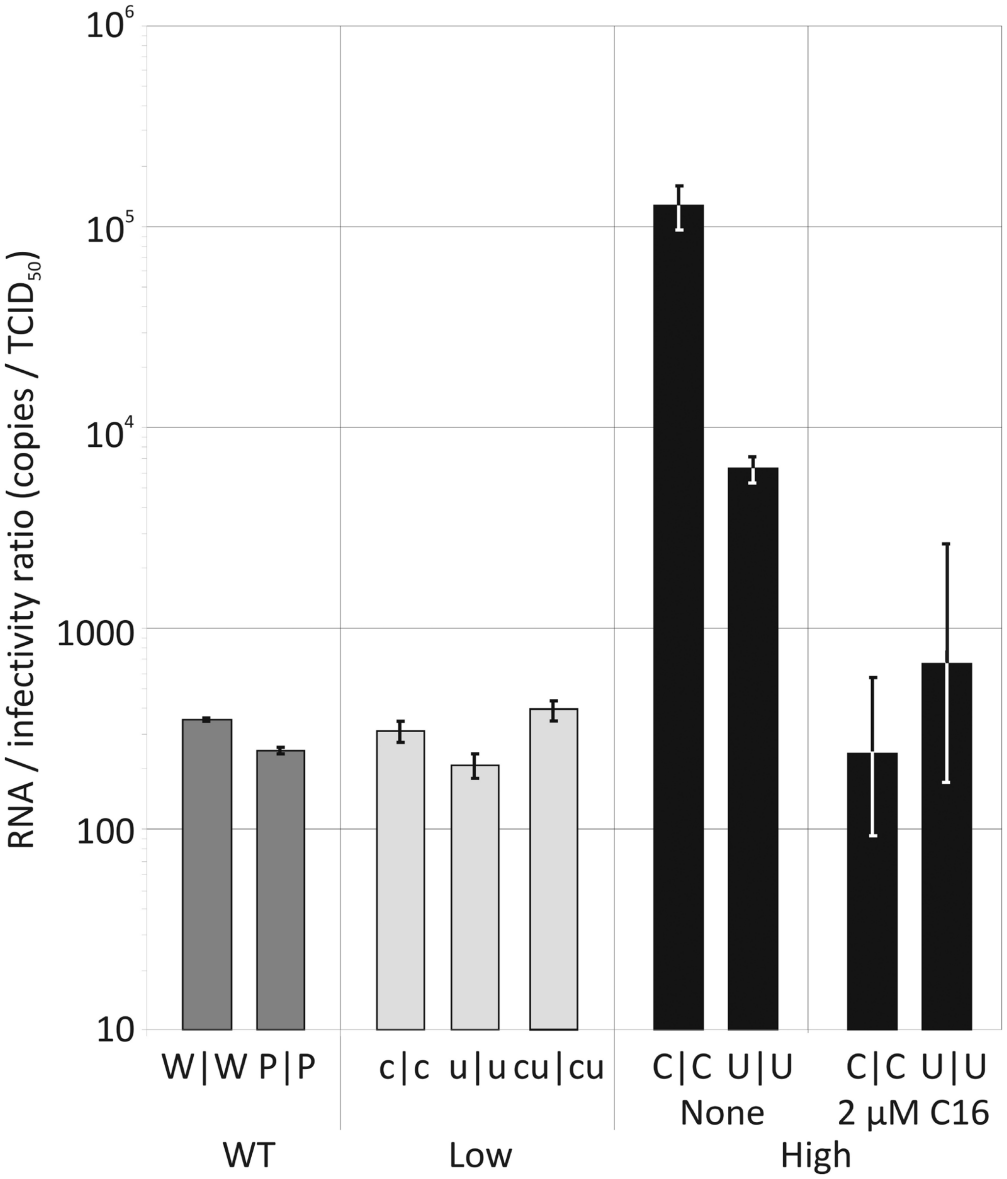
RNA to infectivity ratios of WT and viruses with modified CpG/UpA frequencies. WT and mutant viruses were recovered from RD cells and TCID_50_ titres determined (note log scale). The number of viral genome copies was determined through qRT-PCR and compared with the infectivity titre. Results are the mean and standard error from three separate extractions. RNA/infectivity ratios were additionally calculated for C|C and U|U mutants in the presence of C16.

RNA copy to infectivity ratios were determined by extracting viral RNA from a known infectious titre of each virus, and carrying out qRT-PCR quantification (Figure 2). The RNA to infectivity ratio of the permuted double region mutant, P|P (247 ± 9.2), was similar to that of the WT E7 virus, W|W (354 ± 8.0), indicating that the process of synonymous nucleotide replacement while preserving native dinucleotide frequencies does not affect specific infectivity. In contrast, increasing either the CpG or UpA dinucleotide frequency dramatically increased the RNA to infectivity ratio, with values for the C|C mutant approximately 350 times the WT value and U|U approximately 20 times higher. In contrast, the RNA to infectivity ratios of viruses with reduced CpG and UpA frequencies (c|c, u|u and cu|cu) were comparable with those of W|W (Figure 2).

**Figure 3.**
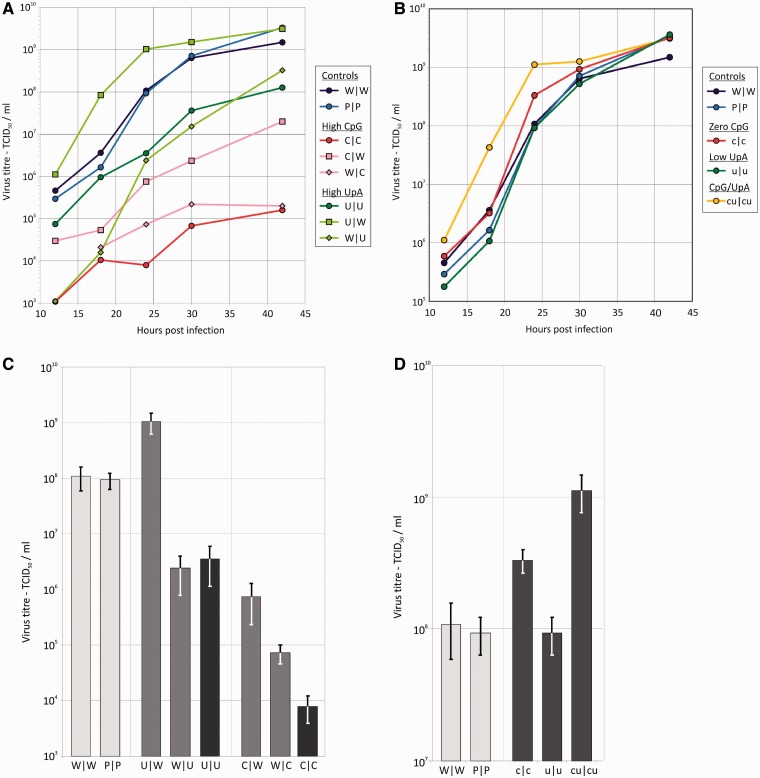
Replication kinetics and titres at 24 h of WT and modified viruses infected at a low MOI. RD cells were infected with E7 WT, permuted (P|P), CpG- and UpA-high mutants (C|C and U|U; **A** and **C**) or CpG- and UpA-low mutants (c|c, u|u, cu|cu; **B** and **D**) viruses at an MOI of 0.01. Infectious titre of supernatants was quantified at indicated time points by TCID_50_ (A and B) and mean titres and SEMs at 24 h. Results are the mean of three biological replicates.

In a multi-step infection using an MOI of 0.01, the growth kinetics of the E7 mutants were compared with that of the WT. Increasing the CpG or UpA dinucleotide frequency caused a severe attenuation of viral replication, resulting in a viral output approximately 7000-fold lower in C|C than W|W after 24 h, and a 30-fold lower output in U|U (Figure 3A andC). Mutant viruses replicated more slowly as well as producing a lower final output of infectious particles. Increasing dinucleotide frequencies in Region 2 was more detrimental to viral replication than Region 1, despite its shorter length (1 kb compared with 1.3 kb), with C|W mutants replicating only 144-fold less than wild type at 24 h, compared to nearly 1500-fold less in W|C. Amongst the UpA-high mutants, replication was actually improved by modifying R1, with U|W consistently showing 10-fold greater replication than wild type while the R2 mutant replicated similarly to U|U. Replication of the (CDLR–)permuted control (P|P) was indistinguishable from wild type, confirming that there were no critical *cis*-acting replication elements within the modified regions. A similar but less marked pattern of replication differences was observed in a single-step replication assay where cells were infected at an MOI of 10 at time zero, including enhanced replication of the U|W mutant (Supplementary Figure S1A).

Mutants with reduced CpG and UpA dinucleotide frequencies compared with the WT level accelerated viral replication (Figure 3B andD). While replication rates and final viral outputs of the CpG-zero (c|c) and UpA-low (u|u) mutants were similar to wild-type and permuted controls, the double mutant in which both CpG and UpA were reduced or eliminated (cu|cu) showed 10-fold higher yield than the wild type at both 18 and 24 h post infection. Replication differences were again less marked in the less-sensitive single-step assay, with similar replication curves between low mutants and controls (Supplementary Figure S1B).

**Figure 4.**
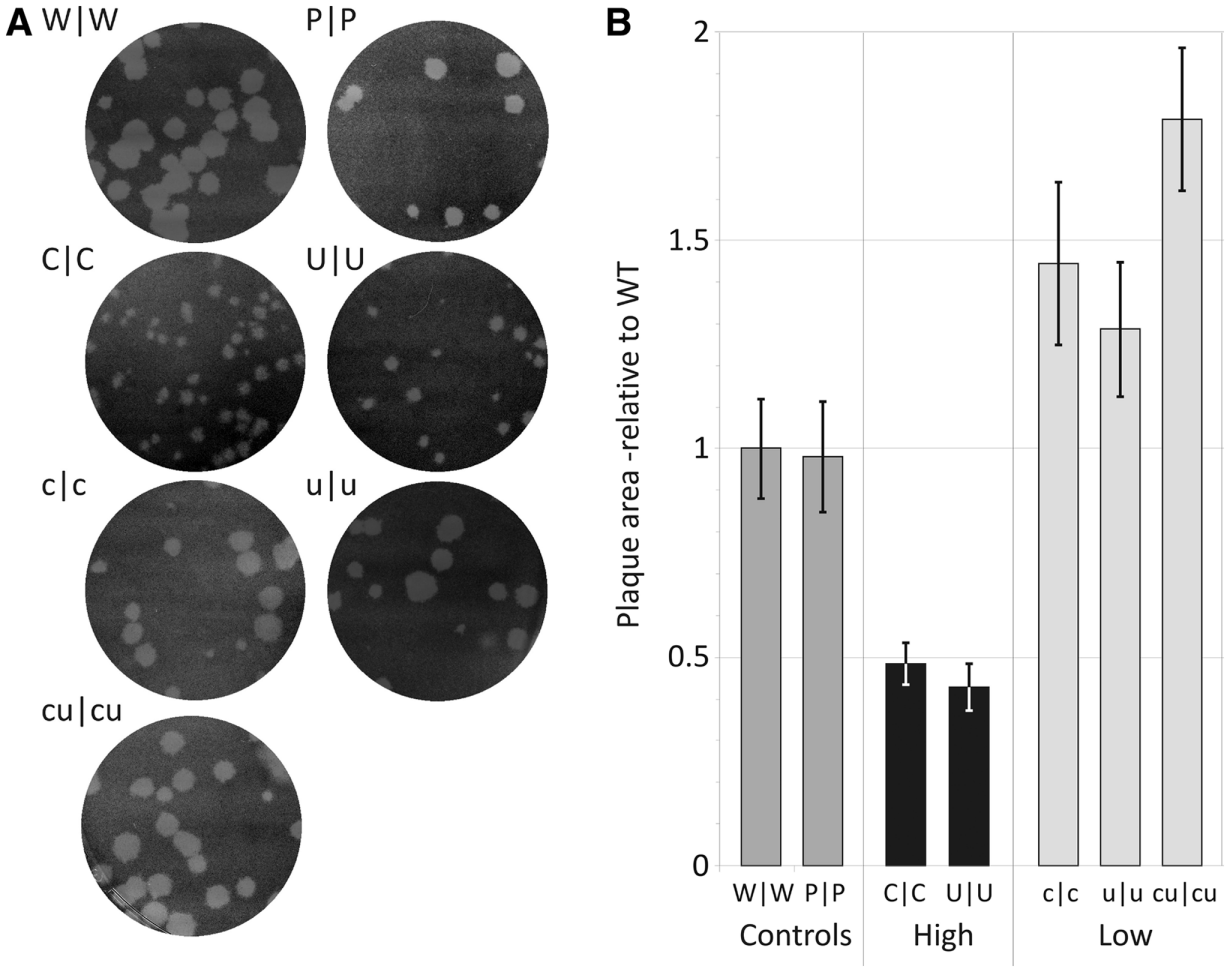
Plaque morphology of E7 WT and double region mutant viruses. RD cell monolayers in 10-cm plates were infected with a similar infectious titre of virus and incubated for 96 h at 37°C. (**A**) Plaque appearance. (**B**) Plaques sizes of WT and mutant E7 viruses calculated from 25 plaques for each virus using ImageJ software (mean values and SEMs relative to WT control shown as bar heights and error bars).

Altering CpG and UpA dinucleotide frequencies also influenced plaque sizes of individual infectious centres created in RD cell monolayers (Figure 4). R1/R2 CpG high and UpA high mutants showed plaques that were respectively 48% (SEM ± 5%) and 43% (± 6%) the size of WT plaques. Areas of UpA- and CpG-low mutants were conversely consistently greater than WT (mean values for c|c: 144% (± 19%); u|u: 129% (± 16%) and cu|cu: 179% (± 17%) of WT size). In contrast, plaques produced by the permuted control, P|P, were indistinguishable to those of WT (98 ± 13%).

**Figure 5.**
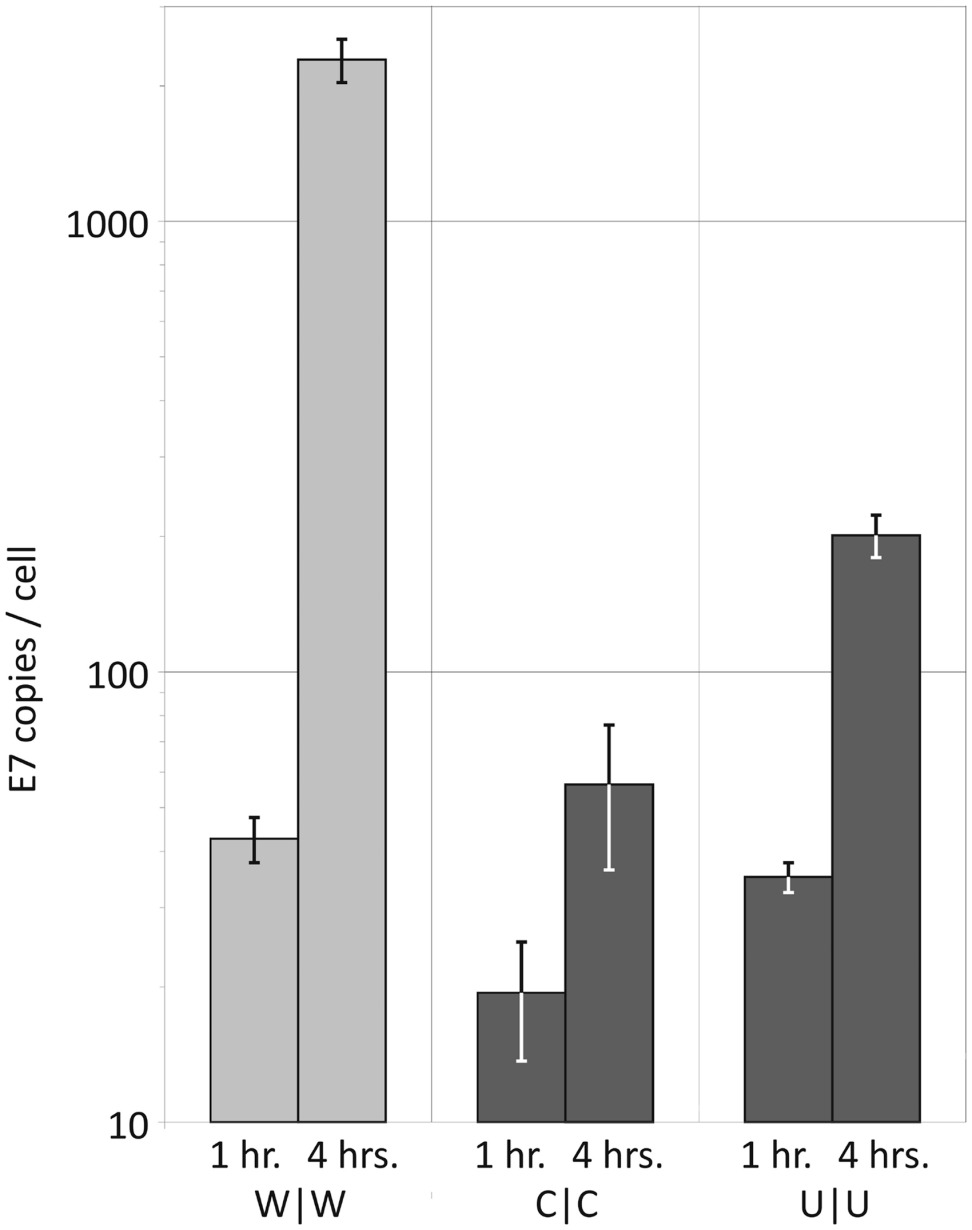
Synchronized infection with equal viral genome copies. RD cells were synchronously infected with 1000 genome copies of WT, R1/R2 CpG-high or R1/R2 UpA-high virus, as calculated using qRT-PCR. Cells were trypsinized and washed 1 or 4 h post infection and the intracellular viral load determined by qRT-PCR. Results are the mean and standard error of three biological replicates.

To determine whether the low RNA/infectivity ratios and reduced replication of CpG- and UpA-high mutants was the result of their failure to initiate an infection cycle or whether there were later restrictions on the generation of infectious virions, RNA copy numbers immediately post-entry were compared with those 4 h after infection. One hour after a synchronous infection with 1000 RNA copies per cell (as determined by qRT-PCR), the number of intracellular viral genome copies was found to be similar between viruses with different dinucleotide compositions, with 42, 19 and 36 RNA copies detected per cell in those infected with WT, C|C and U|U mutants, respectively (Figure 5). However, 4 h post infection, RNA copy numbers of wild-type genome copies increased to 2362 per cell, whilst the RNA copy numbers of C|C and U|U mutants only increased marginally (58 and 207 RNA copies per cell, respectively). The existence of this early impairment has implications in subsequent pathway analyses (see below).

To further investigate the enhancement of replication observed in E7 variants with reduced CpG and UpA frequencies, the effect of dinucleotide changes on the replication of an E7 replicon was investigated. The E7 replicon comprises a monocistronic construct in which structural genes are replaced by a luciferase gene derived from the firefly (*Photinus pyralis*); detection of luminescence enables genome replication to be readily detected and quantified. The luciferase gene in the pRiboE7luc 1.7 kb luciferase gene revealed a strikingly high frequency of CpG (ratio of 1.24; Table 1), consistent with its insect origin (20,22,23). This could potentially attenuate its replication in mammalian cell lines. A replacement luciferase gene was therefore designed in which the CpG ratio was reduced to 0.013 and the UpA ratio to 0.145 (from 0.699), in both cases the lowest values achievable without coding/restriction enzyme site changes, through introduction of synonymous substitutions. The CpG/UpA-low luciferase replicon (designated cu|-|W) was further modified by replacing region 2 with CpG-zero and UpA-low inserts (designated cu|-|c and cu|-|u respectively; the site of Region 1 was occupied by the luciferase gene and indicated as a ‘−’).

**Figure 6.**
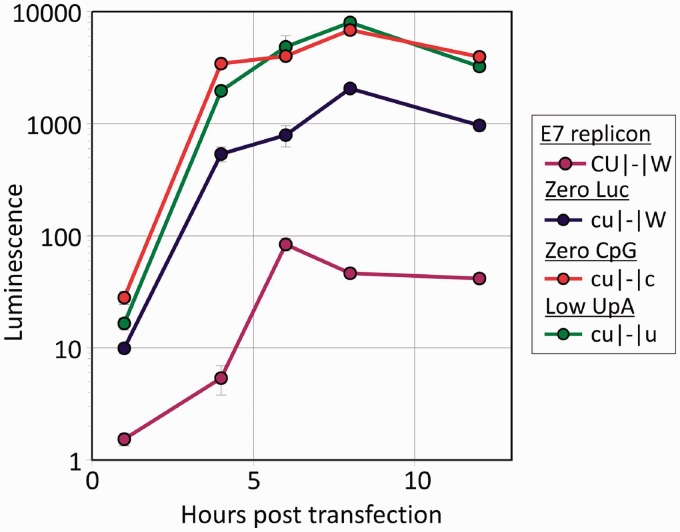
Analysis of luciferase expression driven by E7 replicons with reduced CpG/UpA frequencies. Replicons were generated with reduced CpG/UpA frequencies, based on the backbone *pRiboE7luc* replicon, in which the structural genes of E7 are replaced by an insect luciferase gene. In the cu|-|W replicon the luciferase gene itself was modified to minimize both CpG and UpA frequency; in the cu|-|c and cu|-|u replicons Region 2 was additionally modified to further reduce either CpG or UpA frequency. RNA was generated from replicons and transfected into RD cells. Luminescence was measured relative to the mock-transfected control. Results are the mean and standard error of three biological replicates.

RNA transcripts were transfected into RD cells, and luminescence was monitored over a 12-h time-course (Figure 6). Compared with the original pRiboE7luc replicon, the cu|-|W mutant with the luciferase gene replaced showed a 100-fold increase in relative luminescence as early as 4 h post transfection. The replication of the construct was increased a further 6-fold by replacement of Region 2 with CpG-zero or UpA-low inserts.

### Fitness comparisons of modified viruses using competition assays

**Figure 7.**
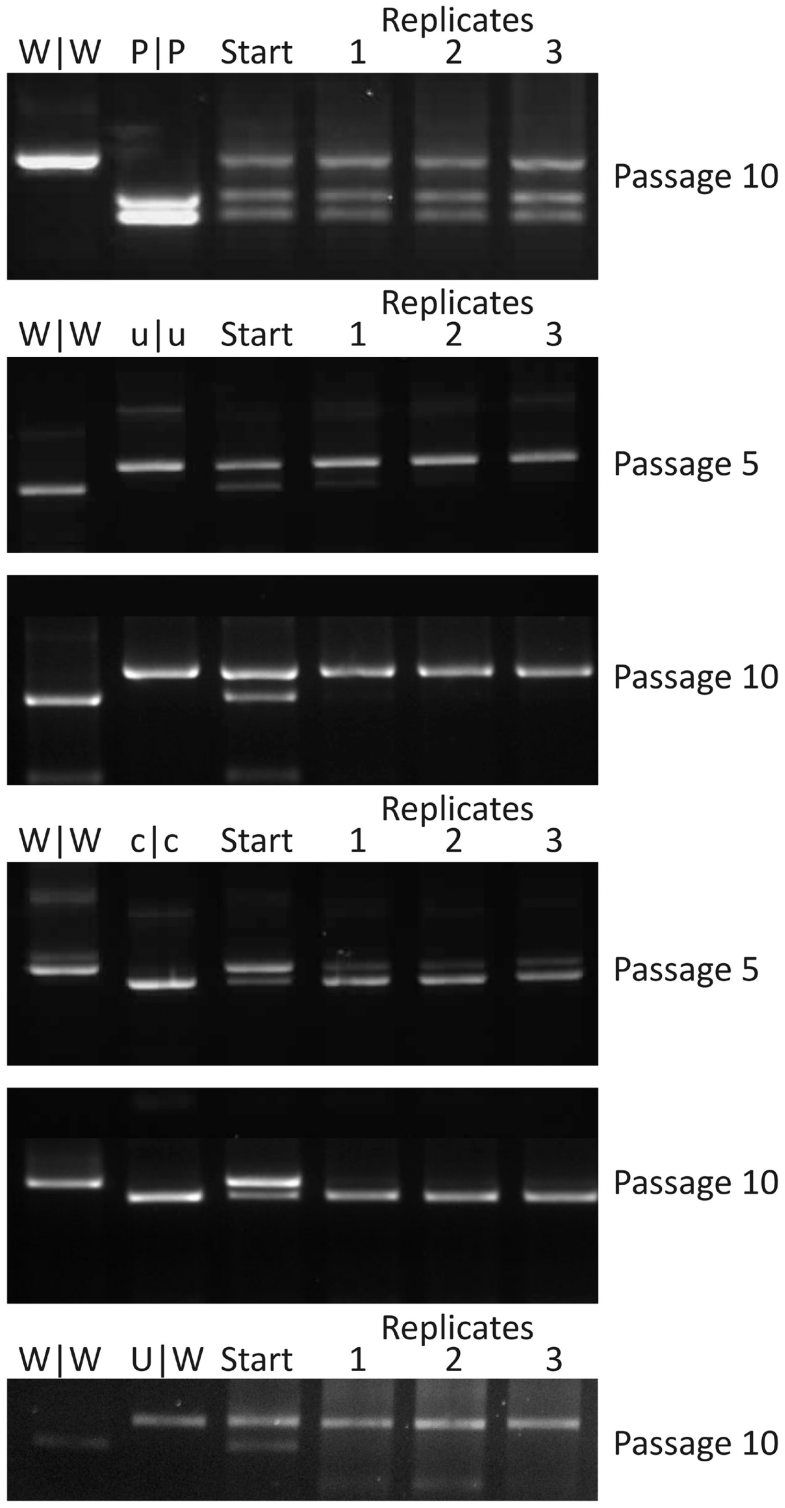
Fitness determination by competition assays between WT and modified viruses. RD cells were infected with an equal MOI of WT and modified virus, and the supernatant serially passaged through cells. RNA was isolated and the composition of each virus determined through selective restriction digests. Images show the virus composition in the starting inoculum and in three biological replicates following passage of CpG- and UpA-low mutants and of U|W.

To confirm the differences in replicative ability of mutants with altered CpG and UpA frequencies in a more sensitive assay, equal MOIs of WT and mutants were co-inoculated onto RD cells and serially passaged at high MOIs between passages. RNA extracted at different passage numbers was amplified across the modified region and cleaved with restriction enzymes that differentiated WT from mutant sequences. As expected, CpG- and UpA-high mutants (C|C and U|U) were rapidly outcompeted by WT virus and were almost eliminated by passage 1, and entirely undetectable at passage 5 (data not shown). Single region mutants (C|W and W|C) were similarly outcompeted by passage 5, consistent with their reduced fitness in the replication assays (Figure 3A and SupplementaryFigure S1A). Reflecting the more marginal phenotype of UpA-high mutants, W|U was eliminated between passage 5 and 10 while U|W outcompeted the wild-type virus by passage 10 (Figure 7). This latter finding confirms evidence for greater replication of this mutant compared with WT in multi- and single-step replication assays.

**Figure 8.**
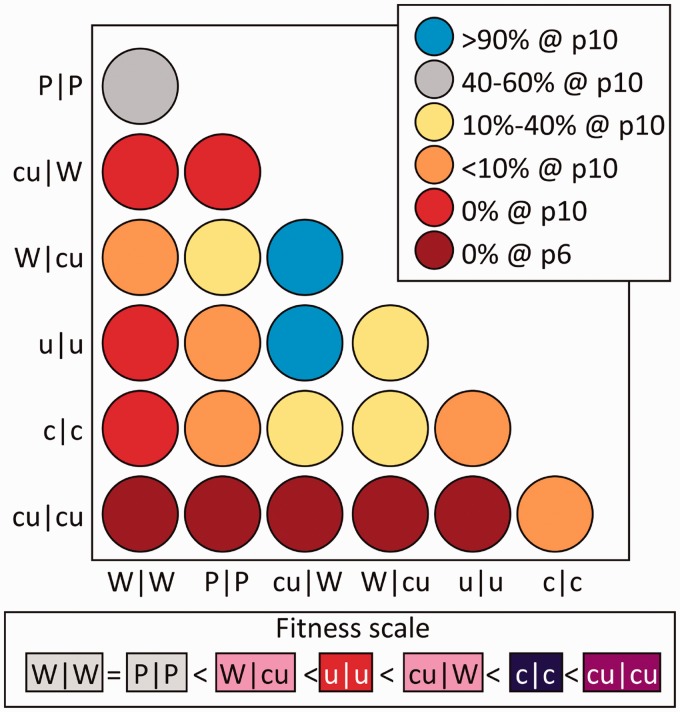
Pairwise fitness comparison between E7 WT and mutant with varying degrees of CpG and/or UpA under-representation. RD cells were infected with an equal MOI of two viruses represented in columns and rows of the matrix and the supernatant serially passaged. The composition of each virus was determined through restriction endonuclease cleavage (see Figure 7) and outcome displayed by colour shading. The key refers to population representations of viruses listed in columns (for example, all but one variants were out-competed by the cu|cu mutant). The fitness ranking deduced from these results is shown underneath the figure.

The same assay format was used to further characterize the unexpected greater replicative ability of E7 mutants with reduced CpG and UpA frequencies; c|c and u|u mutants both outcompeted WT showing at least 80% prevalence after only five passages. By 15 passages, the WT was completely undetectable (data not shown). To investigate this phenomenon further, a series of competition assays were performed in which WT (W|W), the permuted control (P|P) and a range of mutants with differing degrees of CpG and/or UpA underrepresented (cu|W, W|cu, u|u, c|c and cu|cu) were each competed with each other and assayed at passage 6 and 10 (Figure 8). The cu|cu mutant showed the highest fitness, completely outcompeting almost all of the other viruses by passage 6. The c|c mutant ranked second, followed by cu|W. Lowering CpG/UpA frequency in Region 1 was demonstrated to have more effect than in R2, as W|cu was rapidly outcompeted by cu|W as well as u|u. Consistent with the replication assays, reduction in CpG frequency showed a greater effect than that of UpA.

### Mechanism of dinucleotide-dependent replicative fitness differences

The pathway(s) within the cell responsible for the replication phenotypes of viruses with different dinucleotide frequencies is unknown. Investigation of pathways concentrated primarily on measurement of differences between replication between WT and C|C and U|U (high) mutants, as these showed the greatest phenotypic differences in replication assays. Secondly, WT E7 replicated rapidly to high titre in fibroblast cell culture and was evidently highly effective at evading host responses induced by infection. As described below, effective control in many aspects of both recognition and effector pathways precluded detection of phenotypic differences from CpG/UpA low mutants.

**Figure 9.**
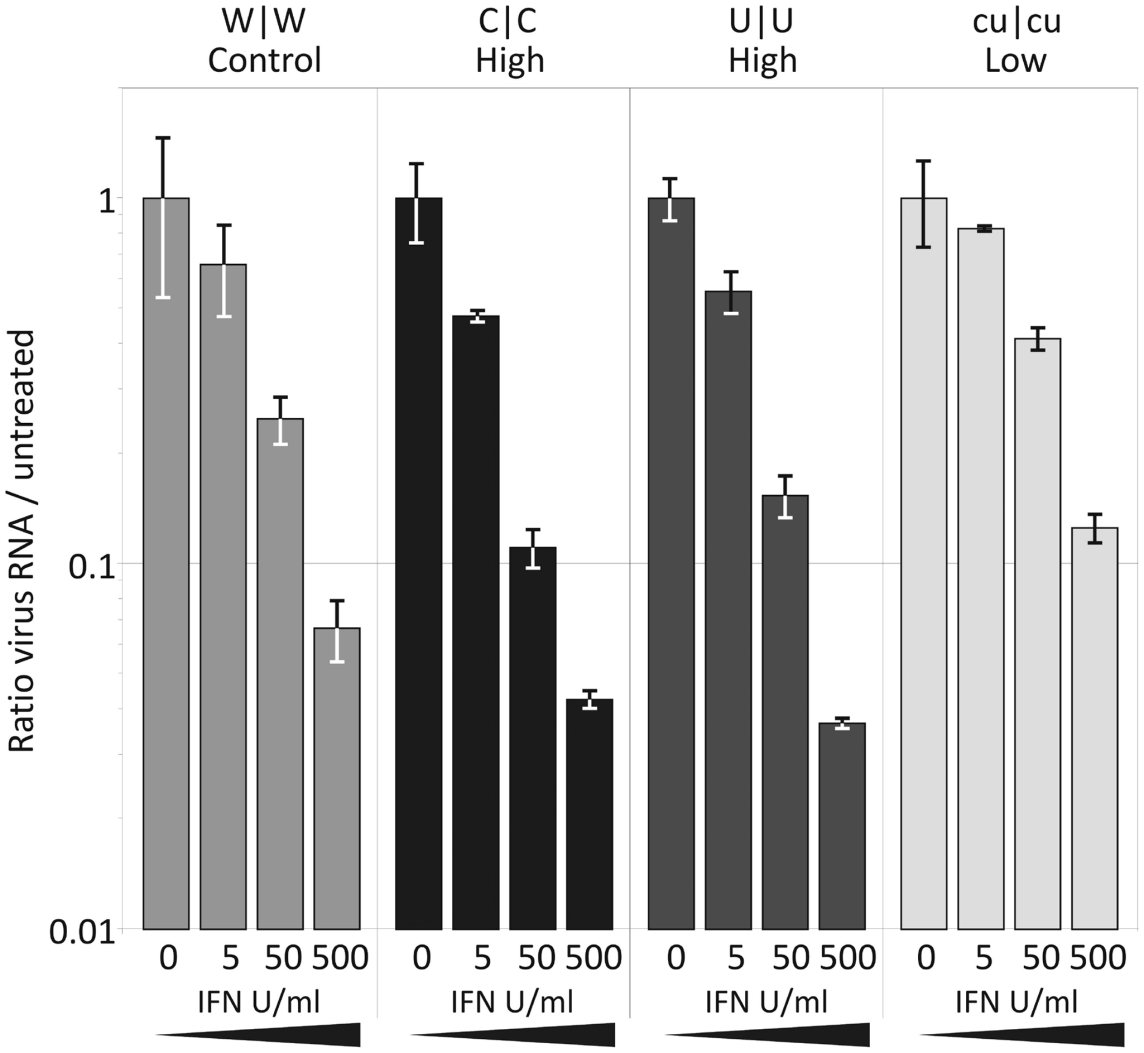
Effect of exogenous IFN-β on viral replication. RD cells were treated with 5, 50 and 500 U/ml human IFN-β, or mock treated, then infected with WT E7, C|C, U|U and cu|cu mutants at an MOI of 1. RNA was isolated after 8 h and viral load determined by qRT-PCR. Results are the mean and standard error of two biological replicates.

Replication differences between WT and mutant with altered dinucleotide frequencies may arise through differences in their susceptibility to IFNβ-coupled cellular defences or through differences in their visibility to pattern recognition receptors (PRRs) that activate the cellular defence response on entry. To investigate the former possibility, susceptibilities of WT and mutant viruses to exogenous IFNβ were determined (Figure 9). Eight hours after infection, WT and mutant viruses showed dose-dependent attenuation of replication, with between 8- to 27-fold reductions in viral RNA levels relative to the mock-treated controls at the highest IFNβ dose. While generally similar to WT virus, the replication of both C|C and U|U (high) mutants showed approximately 2-fold greater susceptibility to IFNβ than WT while the CpG/UpA-low mutant showed approximately 2-fold greater resistance. However, these differences do not account for the 100- to 10 000-fold impairments and 10-fold enhancements in replication respectively observed for these mutants (Figure 3).

A second possibility to explain replication phenotypes is that dinucleotide frequencies influence the susceptibility of viruses to RNA editing by the IFNβ-induced p150 isoform of adenosine deaminase RNA-specific (ADAR1) (24). A greater number of mutations in genomes of CpG- and UpA-high E7 mutants might therefore account for their greatly induced RNA to infectivity ratios (Figure 2). To investigate this, two 500 base regions of the wild type, C|C and U|U mutants were sequenced. Sequences from either the modified Region 1 (nucleotides 1835–2363) or an unmodified region (nucleotides 3723–3241) were amplified and individual population components sequenced by cloning the PCR product and sequencing individual clones. At least 14 clones were sequenced in each region for each virus (Supplementary Table S3). In wild-type E7, no mutations were observed in the total 15.5 kb sequenced. In the C|C virus, three synonymous single-nucleotide changes were observed in the total 15 kb sequenced, while in U|U a total of 15.5 kb revealed one synonymous change and one U→C substitution, converting a methionine residue into a threonine. Although the amount of sequence data obtained was limited, the predominantly synonymous mutations observed in C|C and U|U mutants do not suggest any substantially greater susceptibility to ADAR1-mediated mutation, and this would be unlikely to be responsible for their dramatically impaired replication.

**Figure 10.**
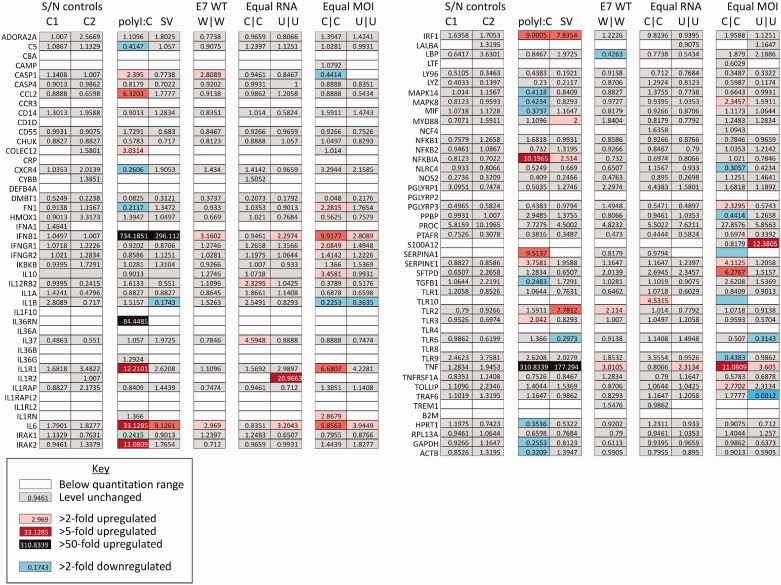
Analysis of gene expression in cells infected with WT E7 and mutants. RD cells were infected with E7 at an MOI of 10 and changes in expression at 8 h of a range of innate and adaptive immune response genes determined using a PCR Array. Responses were compared with those induced in cells infected by high CpG- and UpA mutants at equal copy number to WT or equal MOI. Parallel infections with Sendai virus (SV) and transfection of poly-I:C were performed. Non-specific induction of genes was controlled for by parallel assays of cells exposed to u/v inactivated supernatant corresponding in amount to that added in the equal copy number (C1) and equal MOI (C2) assays.

Another possibility for the different phenotypes of E7 dinucleotide mutants is that they differentially activate the IFNβ response in infected cells. To investigate the cellular response to infection, the expression of 90 innate immune response genes was analysed following infection with E7, C|C or U|U viruses. WT E7 induced a minimal IFNβ response at 8 h with transcript levels at the limit of detection, a similarly unaltered level of the early interferon-stimulated genes (ISGs) 15 and 56, and small changes in expression of a total of six genes from the 90 immune response genes (Figure 10 and Supplementary Figure S2). Of these, five were up-regulated 2- to 5-fold and one was down-regulated. The response was quantitatively and qualitatively much weaker than in cells transfected with poly-I:C or infected with Sendai virus (Figure 10), where there were large increases in the expression of IFN-β and a range of other innate response genes.

Similarly, inoculating cells with infectious doses of C|C and U|U mutants equivalent in RNA copy number to those of W|W (i.e. MOIs 100- to 1000-fold lower than WT) induced no IFNβ or ISG responses and minimal changes in gene expression of other genes in the PCR array (>2- and <5-fold induction of three and four genes, respectively; Figure 10 and Supplementary Figure S3). This demonstrated that RNA genomes with increased dinucleotide frequencies entering the cell equivalently to WT (see above) activated only a minimal cellular response. However, infecting cells with U|U and particularly C|C mutants at equal MOI to WT induced a higher IFNβ response, which was also reflected by a greater number of changes in gene expression in the PCR array. Infecting with the C|C mutants led to 12 genes being up-regulated (and 5 down-regulated).

**Figure 11.**
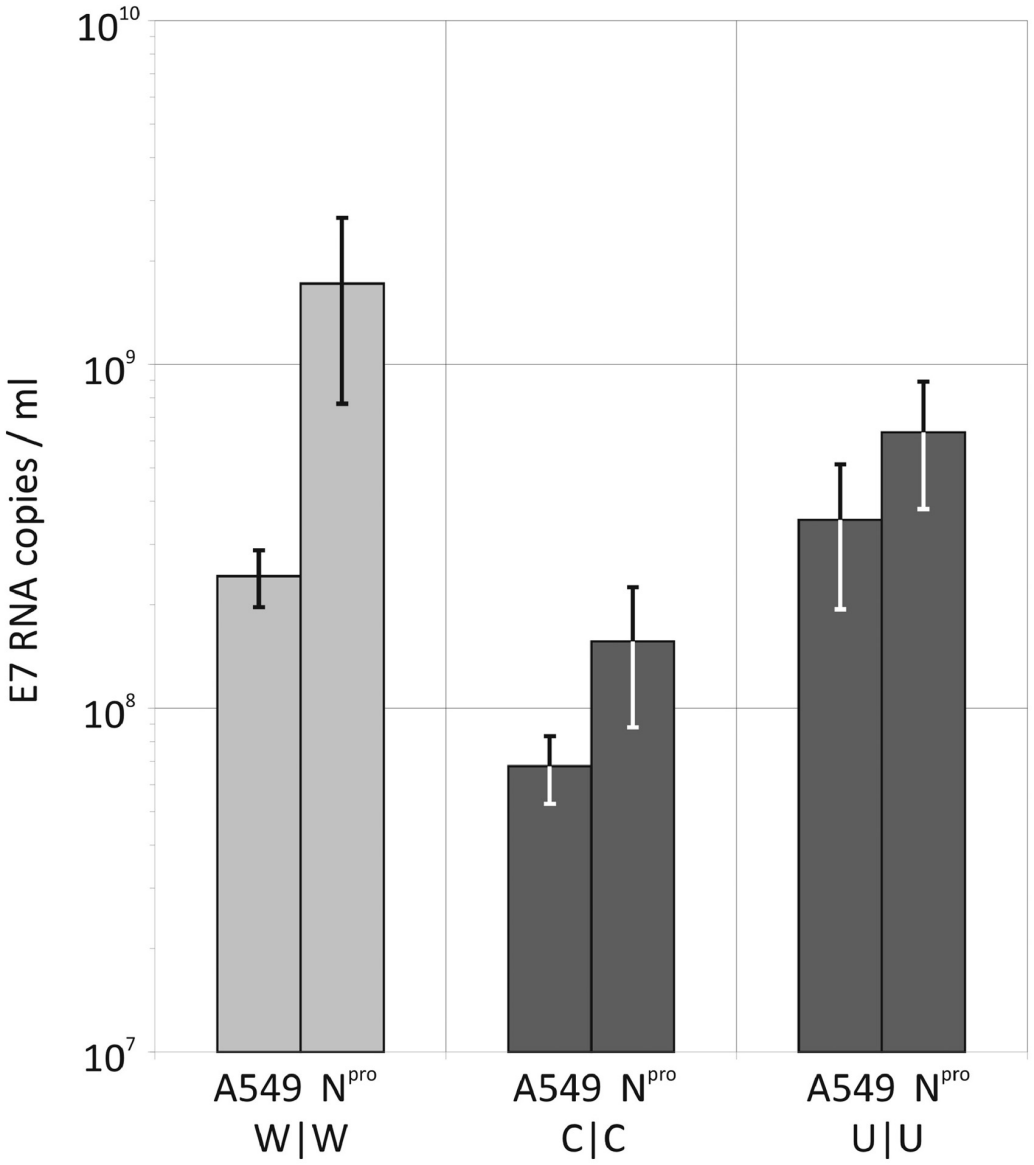
Replication of WT and dinucleotide-modified viruses in an IRF3-blocked cell line. A549 cells expressing the viral protein N^pro^ and non-expressing control cells were infected with WT, C|C (R1/R2 CpG-high) and U|U (R1/R2 UpA-high) at an MOI of 0.01. RNA was extracted from cell culture supernatant at 24 h and viral load determined by qRT-PCR. Results are the mean and standard error of three biological replicates.

To investigate potential signalling pathways that were differentially activated by viruses with differing dinucleotide frequencies, the replication of E7 and mutant viruses (C|C and U|U) was measured in cells in which expression of specific signalling component was inhibited. Interferon regulatory factor 3 (IRF3) is a central regulator of type I IFN induction in response to the detection of viral RNA in the cytoplasm or endosomes, and its signalling is blocked by the viral N^pro^ protein from classical swine fever virus (25). Both mutant and WT viruses inoculated at equal MOIs replicated to higher levels in cells expressing N^pro^ compared with the parental A549 cell line, with 8-fold increases in WT virus and 3- to 4-fold in C|C and U|U (Figure 11). Replication of E7 is therefore at least partially controlled through activation of the IRF3 signalling pathway even though IFNβ and other cellular responses are highly restricted. However, there was no evidence that IRF3-signalled inhibition of virus replication was differentially activated by mutants with elevated CpG and UpA dinucleotide frequencies; fold increases in replication were actually less than those displayed by WT E7.

**Figure 12.**
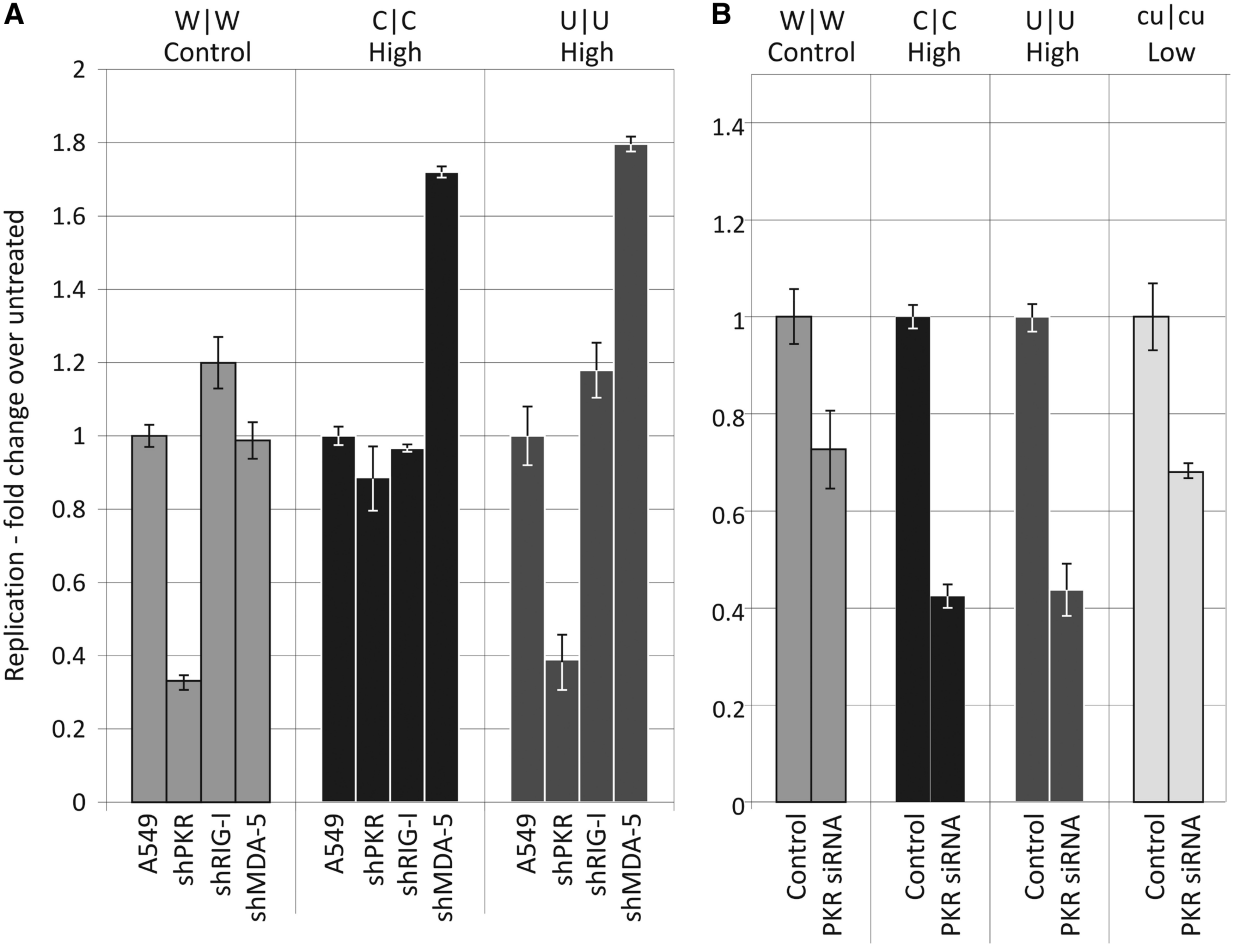
Effect of inhibiting PKR, RIG-I and MDA-5 expression on E7 replication. (**A**) Comparison of virus replication in control A549 cells at 8 h compared with that in cells expressing shRNAs targeting PKR, RIG-I and MDA-5 (reduction in mRNA levels achieved is shown in Supplementary Figure S3). (**B**) RD cells were pre-treated for 24 h with an siRNA directed against PKR and infected with WT virus or CpG-high, UpA-high or CPg/UpA-low mutants at an MOI of 0.03. RNA was isolated after 22 h and intracellular viral loads determined by qRT-PCR. Results are shown relative to the replication rate in cells treated with a control (validated non-target) siRNA.

IRF3 is part of a signalling pathway that is activated upstream by the cytoplasmic dsRNA sensors RIG-I and MDA5. Another PRR that is potentially responsible for differential recognition of infecting viruses with differing dinucleotide compositions is PKR. To investigate their roles in recognition, replication of W|W, U|U and C|C mutants was determined in A549 cells expressing shRNAs targeting each PRR (Supplementary FigureS3). Down-regulation of RIG-1 had no effect on the replication of WT or mutant E7 variants, while the replication of C|C and U|U mutants (but not WT E7) was marginally increased (2-fold) in cells expressing the MDA-5 shRNA (Figure 12A). In contrast, down-regulation of PKR had a partial inhibitory effect on the replication of WT and U|U mutant E7 variants, but little or no influence on C|C mutant replication.

We investigated this latter observation further by inhibiting PKR expression in RD cells by transfecting them with a PKR-specific siRNA to PKR. These down-regulated PKR mRNA and protein expression by >80%, irrespective of whether infected by E7 or not (Supplementary Figure S4). Under these conditions, the replication of WT and C|C, U|U mutants was consistently reduced compared with that in cells pre-treated with the control siRNA as was the replication of the cu|cu mutant, which we additionally tested (Figure 12B).

**Figure 13.**
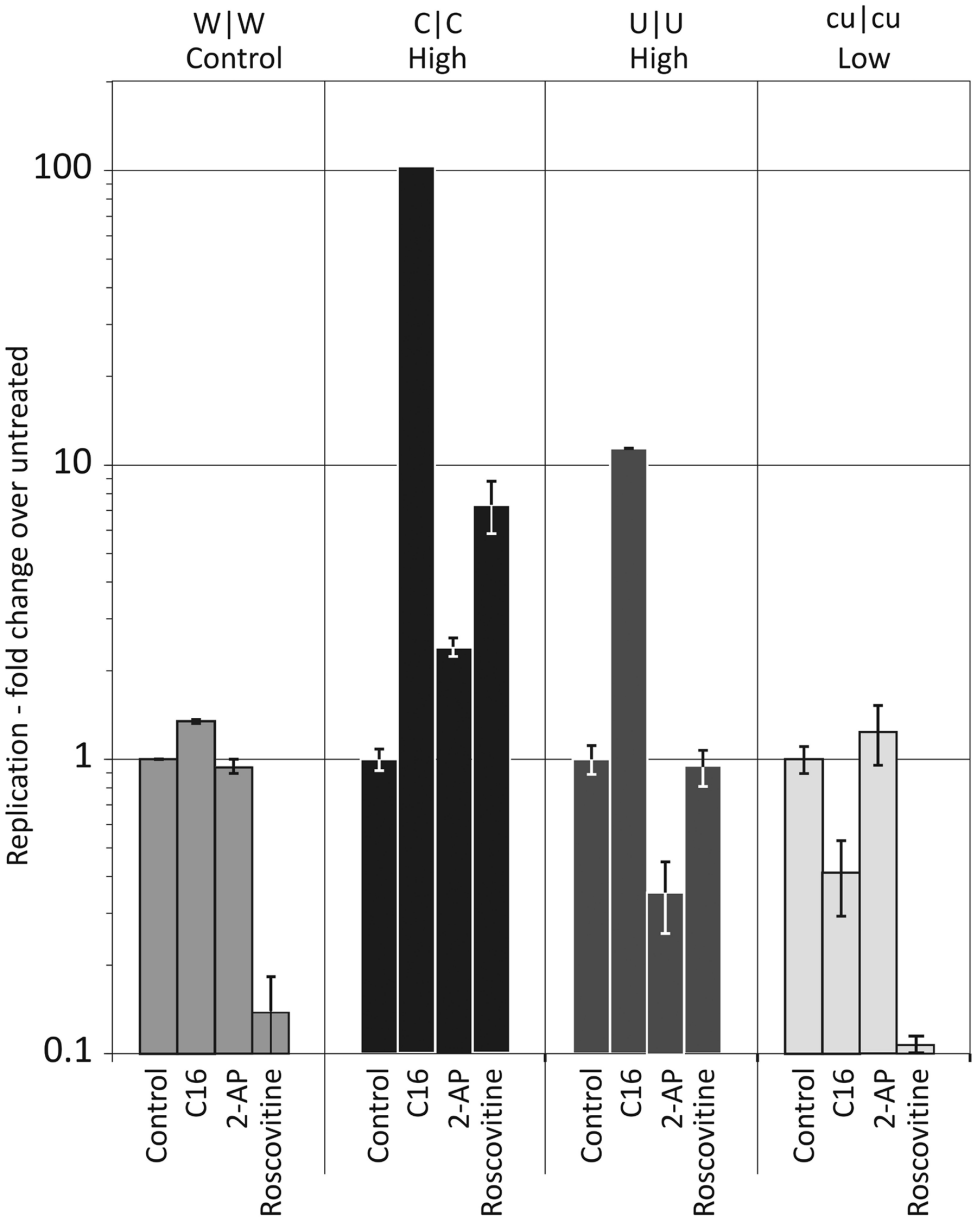
Effect of the kinase inhibitors, C16, 2-AP and Roscovitine on E7 replication. Cells were pre-treated for 45 min with 2 μM C16, 5 mM 2-AP or 40 μM Roscovitine and infected with WT virus or CpG-high, UpA-high or CpG/UpA-low mutants at an MOI of 0.1. RNA was isolated at 24 h, and intracellular viral loads were determined by qRT-PCR. Results are shown relative to the replication rate in cells untreated cells (note log scale).

**Figure 14.**
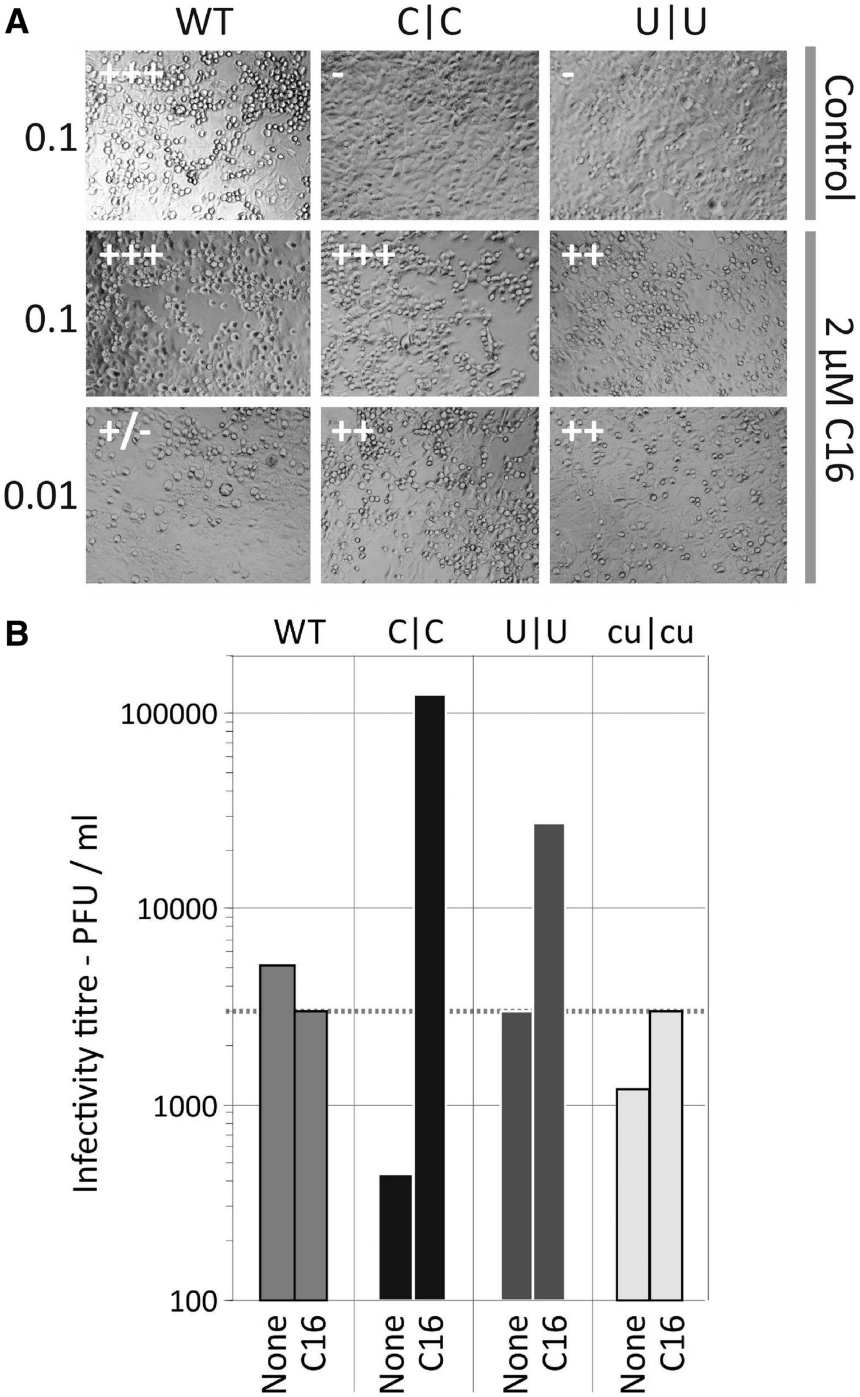
Influence of the PKR inhibitor E7 cytopathology and infectivity. (**A**) Cytopathology of WT, C|C and U|U mutants in RD cells infected at MOIs of 0.1 and 0.01 in 2 μM C16 or untreated control. Monolayers of untreated cells remain intact on infection with C|C and U|U mutants while those treated with C16 display a complete CPE even at low MOI. (**B**) Infectivity determination of pre-titred virus stock supernatant (3000 PFU/ml) of WT virus or CpG-high, UpA-high or CPg/UpA-low mutants in the presence of C16. The enhanced infectivity of C|C and U|U mutants restored the RNA/infectivity ratios to those of WT virus (Figure 2).

Although specific knockdown of PKR expression by shRNA and siRNA typically inhibited replication of E7 and its mutants, treatment of cells with the kinase inhibitor C16 dramatically enhanced virus replication of mutants with increased frequencies of CpG and UpA dinucleotides (Figures 13 and 14). Treatment of cells with C16 led to 10- to 100-fold increased in the replication of C|C and U|U mutants, while the replication of WT and cu|cu (CpG/UpA-low) mutants was relatively unaffected (Figure 13). We extended this study to determine the cytopathology of selected mutants. Infecting cells with WT virus at an MOIs of 0.1 produced visible pathology after 22 h, and limited cytolysis at an MOI of 0.01 (Figure 14A). This was unaffected by the addition of C16. In marked contrast, untreated cells infected with C|C and U|U mutants showed no cytopathology over the experimental interval while those treated with C16 showed complete CPE at an MOI of 0.01. C16 treatment similarly increased the infectivity of C|C and U|U mutants (Figure 14B). Virus stocks containing 3000 PFU/ml, as measured previously on untreated RD cells, were re-titrated in a quantal infectivity assay with six replicated at serial 10-fold dilutions and infectious titres determined by probit analysis. C16 treatment had no effect on the infectivity of WT or cu|cu E7 variants, while infectivity was enhanced 10-fold (U|U) and nearly 1000-fold (C|C) in mutants with increased dinucleotide frequencies (Figure 14B). Using these revised infectivity titres, C|C and U|U mutants showed equivalent RNA/infectivity ratios to WT virus in the presence of C16 (Figure 2). The attenuating effect of increased CpG and UpA frequencies on E7 replication is thus entirely reversible by C16; the reduced infectivity of high CpG/UpA mutant stocks is clearly not because the virus is intrinsically defective but because the cell is better able to prevent infection by these mutants than by WT virus.

Although C16 has been considered to be a specific inhibitor of PKR (26), its effect on the replication of GpG- and UpA-high mutants was inconsistent with the lack of similar replication enhancement using sh- and siRNAs directed against PKR mRNA (Figure 12). The possibility of an off-target effect of C16 was further suggested by the lack of an equivalent affect on the replication of C|C and U|U mutants by 2-AP that also inhibits PKR phosphorylation (Figure 13). Documented additional targets of C16 include the cyclin-dependent kinases, CDK2 and CDK5 (27). To investigate whether these unintended actions of C16 were influencing E7 replication, we determined the effect of a well characterized inhibitor of cyclin kinases, roscovitine, on the replication of WT and mutant variants. Cells infected with WT and cu|cu E7 variants showed nearly 10-fold reduced E7 replication compared with untreated cells while the replication of U|U replication was unchanged and C|C substantially enhanced (≈9-fold). As observed with C16 treatment, roscovitine induced visible cytopathology in C|C-infected cells while none was apparent in controls (data not shown). Increased C|C replication occurred despite a general reduction in cellular gene expression in treated cells (including a 3-fold reduction in the housekeeping gene, GAPDH).

## DISCUSSION

Although decreases in virus fitness and replication by increasing CpG and UpA frequencies in RNA virus genomes has previously been described for poliovirus, this study is the first to demonstrate the converse, that decreasing frequencies of these dinucleotides significantly increases virus replication. This remarkable finding raises profound questions about the nature and direction of evolutionary selection operating in enteroviruses and potentially each of the many other RNA viruses that show similarly suppressed CpG and UpA frequencies (10–12). The study additionally demonstrates that while E7, along with other enteroviruses, is highly effective at suppressing IFNβ induction and activation of host cell defences, cells are nevertheless able to recognize and restrict replication of mutants with differing dinucleotide frequencies. The findings point towards a novel mechanism for recognizing foreign RNA that prevents establishment of virus replication at an early time point after virus entry.

### Relationship between virus replication and CpG and UpA frequencies

Increasing the frequency of CpG or UpA dinucleotides in E7 resulted in severe viral attenuation, characterized by reductions in replication rate, smaller plaque areas, low particle to infectivity ratio and a low competitive fitness relative to WT E7. These findings are consistent with replication defects observed in poliovirus with elevated CpG and UpA frequencies in the capsid gene (17,28,29). The 2009 study by Burns *et al.* provided a convincing demonstration that the previously reported attenuating effect achieved by codon de-optimization (28) was secondary and proportional to increases in CpG and UpA frequencies rather than a slowing of virus replication by inefficient translation. Moreover, it was shown that changes in the capsid to conventional measures of translation efficiency (CAI, Enc, CPB) correlated poorly if at all with replication rate, in contrast to direct correlations with changes in CpG and UpA dinucleotide frequencies.

Construction of mutants that increased bias towards unfavourable codon pairs (19) were remarkably effective at reducing poliovirus replication but again these also substantially increased CpG and UpA dinucleotide frequencies. In their study, however, the PV-Min mutant contained an observed to expected frequency of CpG of 1.34 (compared with 0.63 of the WT sequence) and 1.36 for UpA (WT: 0.74) that might similarly account for the observed differences in replication phenotypes rather than translation effects. These indeed approach levels in the CpG-high and UpA mutants constructed in the current study (Table 1). Contrastingly, their attenuation was inconsistent with the modest 50–70% measured reductions in translation efficiencies of the PV-MinXY and PV-MinZ mutants. Indeed, the failure to recover any infectious virus from the whole capsid mutant, PV-Min seems incompatible with a measured 25% translation efficiency. A previous study in which translation efficiency of poliovirus 5’UTR mutants was reduced to 12–23% of WT levels showed an approximately proportionate (1–1.5 log) reduction in replication kinetics (30), quite different from the replication impairment observed in PV-MinXY, PV-MinZ and PV-Min. Together, these findings concur with the evident lack of correlation between CAI, Enc and CPB values in the E7 insert sequences (Table 1) and their replication phenotypes when inserted into E7. As examples, CAI values for CpG-zero sequences were almost identical to WT E7 while their insertion enhanced replication. CpG- and UpA-high sequences actually had higher CAI values yet showed an impaired replication phenotype. There was a similar lack of association with Enc and CPB measures and phenotype.

Our findings imply a fundamental role of dinucleotides in replication ability of enteroviruses and potentially other RNA viruses that show evidence for suppression of CpG and UpA frequencies. We would argue that their phenotypic effects have to be included and allowed for in viral evolutionary studies such as those measuring often subtle effects of codon usage or codon order on virus replication. A case in point relates the concept of evolutionary ‘robustness’ of RNA viruses, based on the hypothesis that the evolutionary fitness of viral populations is dependent on shape of the fitness landscape around a consensus wild-type master sequence (31–33). A recent investigation of the effect of different fitness landscapes compared WT poliovirus with two mutants in which codon order, but not usage, was altered (34). These were constructed using capsid region sequences in which codon pair frequencies were skewed towards those most favoured in human coding sequences (PV-Max) and one where codon order was randomly permuted while retaining CPB (PV-SD). Importantly these had the same CAI and Enc values to WT sequences (and in the case of SD, an identical CPB); both showed equivalent translation efficiencies in *in vitro* translation assays.

As expected from their different positions in ‘sequence space’, the mutants showed different mutational spectra on passaging with Ribavirin. However what was unexpected was their replication phenotypes, with PV-SD showing lower fitness but PV-Max greater fitness in competition assays with WT. The behaviour of PV-Max is particularly intriguing as, in common with PV-SD, there were hundreds of sequences changes from the WT sequence and likely far from an optimal ‘robustness’ as conceptualized in the study. We suggest a contributory factor or alternative explanation for the replication phenotypes was the unintended alteration of dinucleotide frequencies in the mutant sequences consequent to permutation of codon order. The PV-SD mutant (unlike CDLR in the current study) showed increased CpG and UpA frequencies from 0.60−> 0 88 and 0.8−>0.91 respectively, changes that are consistent with its observed modest fitness defect in competition against WT and lag in replication kinetics. Conversely, optimization for CP frequencies in PV-Max contrived to decrease CpG and UpA frequencies to 0.40 and 0.60 and which potentially account for its slight fitness advantage over WT in competition assays, although to a proportionately lesser extent than the cu|W and cu|cu E7 mutants in the current study. Our study additionally provides direct evidence against the hypothesis for robustness. The E7 codon permuted sequences (P|P) generated by the CDLR algorithm in the current study showed 251 sequence differences from WT yet preserved the exact dinucleotide frequencies of the WT sequence. Although clearly also residing in a quite different ‘sequence space’, it showed identical replication fitness to WT, being present in equimolar amounts to WT even after 10 passages in a competition assay format (Figure 7).

Competition assays with WT E7 and a range of CpG- or UpA-low mutants in regions 1 and 2 showed a close correlation between fitness and numbers of CpG and UpA dinucleotides removed (Figure 8). The proposed fitness ranking additionally demonstrates that both dinucleotides participate in the fitness enhancement. Removal of CpG/UpA dinucleotides in Region1 had a greater fitness effect than Region 2. This likely reflects natural compositional differences between the two regions; the region 1 WT sequence showed a observed/expected CpG frequency of 0.73, over twice that of Region 2 (0.32). From the first region, 56 CpG dinucleotides were removed to generate the CpG-zero mutant, over twice that of Region 2 (*n* = 21). Compositional differences also explain why Region 2 CpG-high mutants showed greater fitness reductions that Region 1, as CpGs are already over-represented in the latter part of the genome.

As a possible contributory factor to the otherwise unexplained greater replication of U|W compared with WT (Figure 3A andC), generating this mutant led to 12 CpG dinucleotides being removed from Region 1 (Table 1). Based on current understanding from the phenotypes of other mutants, this seems insufficient to fully account for the fitness gain. However, we know little about whether specific thresholds for dinucleotide removal exist; specifically, are fitness gains associated strictly proportional to the number of CpG and UpA dinucleotides removed, or could relatively small reductions in frequency display the full phenotypic effect? As a broader question that relates to thresholds, does removing (or adding) CpG and UpA dinucleotides in two 1000- to 1200-nt regions have the same effect on replication as adding/removing the same number over the whole genome. Whether extending the region of dinucleotide removal to the rest of the genome (excluding critical replication determinants) would lead to further proportionate increases in replication is similarly uncertain and is an obvious priority in further studies.

Until we know more about the sequence motifs responsible for the differential recognition of sequences of different dinucleotide frequencies, these questions will remain unresolved. However, the existence of thresholds can be determined through fitness determination of mutants with differing degrees of CpG or UpA removal. These investigations are currently planned and will necessitate a more sophisticated mutagenesis algorithm that introduces additional mutations to compensate for those introduced to add or remove targeted dinucleotides. The method can maintain exact mononucleotide frequencies and protein coding and, to the greatest allowable extent, frequencies of non-targeted dinucleotides.

Finally, changes in CpG frequency showed a greater effect on viral replication than changes in UpA levels, being both more beneficial to replication when lowered, and more detrimental when raised. When competed directly, the double region CpG-low mutants were fitter than their UpA-low mutant counterparts. These findings are consistent with those in poliovirus where CpG-high mutants also exhibited a more severe attenuation than UpA-high mutants (17); selection to remove CpG dinucleotides was similarly substantially greater than against UpA during serial passage of mutated viruses (28). Indeed, dissimilar patterns of CpG and UpA suppression amongst viruses and host genomes (10,11,31) point to different selective pressures acting upon each dinucleotide.

Irrespective of the underlying mechanism by which removal of CpG and UpA enhances RNA virus replication, the findings may potentially be exploited for enhancing replication of viruses or other genomes recovered by reverse genetics for research, therapeutic, vaccine or other purposes. Changes in the luciferase gene alone enhanced reporter gene expression in the E7 replicon by >10-fold even though constructs of this type containing luciferase and other reporter with similarly elevated CpG frequencies have been in use for many years without any idea that their replication or reporter gene expression was potentially suppressed. High CpG frequencies may similarly limit the expression of transgenes and DNA vaccines (35); as with codon pair manipulation, the observed enhancement of expression of mammalian ‘codon-optimized’ versions of reporter gene derived from non-vertebrates may indeed be secondary to reductions in CpG frequencies. Finally, large scale removal of CpG and UpA dinucleotides from viruses used as seed stocks for inactivated virus production (including influenza A and B viruses and poliovirus) may increase cell or egg culture virus yields substantially. Experimental investigation of green fluorescent protein transgene expression in mouse embryos and of influenza production in cell and egg culture using synthetic sequences in which CpG and UpA have been largely eliminated from coding sequences is currently underway.

### Mechanism of CpG and UpA recognition

In common with other RNA viruses infecting mammals and birds, enteroviruses and other picornaviruses have evolved numerous strategies to evade the antiviral effect of the innate host response to infection. The effectiveness of E7 in blocking these cellular response pathways is attested by the virtual absence of IFNβ mRNA synthesis on infection with high MOIs of WT virus and the minimal up-regulation of other innate immune response genes (Figure 10 and Supplementary Figure S2). Evasion strategies likely resemble those of other enteroviruses that are known to deploy a range of signalling blocks mediated through proteolytic cleavage or inhibition of signalling by RIG-I, MDA-5, TIR-domain-containing adapter-inducing interferon-β (TRIF), mitochondrial antiviral signaling protein (MAVS) and the IFNβ receptor, IFNAR1 (36–39), typically by the virally encoded proteinases 2A and 3C. The enterovirus 2A proteinase additionally cleaves the translation initiation factor, eIF4G and turns off cap-dependent translation of cellular mRNAs (40). These strategies exert a powerful blunting effect on innate as well as acquired immune responses in the host, as exemplified in hepatitis A virus through cleavage of both TRIF and MAVS and prevention of both TLR- and cytoplasmic PRR signalling (41).

Because E7 effectively paralyses IFNβ-coupled cellular responses in infected cells, it is most unlikely that the differential replication ability of E7 mutants with altered dinucleotide frequencies is mediated through differences in susceptibility to IFNβ-coupled defence pathways. Observationally both WT and CpG- and UpA-high mutants were similarly susceptible to exogenously added IFN (Figure 9) and E7 mutants showed little evidence for greater RNA editing by the IFNβ-activated p150 isoform of ADAR-1 (24) that might otherwise have potentially accounted for their marked differences in RNA/infectivity ratios (Figure 2). The restoration of specific infectivity of C|C and U|U variants by C16 to WT levels (Figure 2) indeed demonstrates that these mutant viruses are not intrinsically defective.

Differences in viral fitness are therefore likely to arise due to differential activation of the host innate immune system, a hypothesis supported by the observation of almost complete attenuation of replication very early after infection of RD cells with C|C and U|U mutants. These were able to enter cells within an hour but subsequently either failed to initiate replication or showed dramatically reduced genome amplification compared to WT E7 (Figure 5). Candidate PRRs that conventionally recognise infection by RNA viruses include RIG-I and MDA5 although ascribing a protective role against E7 is problematic. For the former, recognition requires RNA sequences containing 5’ triphosphate groups while MDA5 requires long dsRNA sequences created during virus replication (42–44); neither of these PAMPs are present immediately after virus entry. Furthermore, their signalling is likely to be substantially inhibited by viral proteinases that cleave MAVS, TRIF and potentially also the PRRs themselves (36–39). Furthermore, neither RIG-I nor MDA5 seem capable of inducing responses in the absence of virus replication and synthesis of dsRNA replication intermediates (45). Finally, inhibition of RIG-I and MDA5 expression led to, at best, minimal increases in E7 replication (Figure 12). This strongly suggests that neither of these conventional PRRs are primarily responsible for the block on replication of high CpG or UpA mutants.

Remarkably, however, treatment of cells with C16, generally considered to be a PKR-specific inhibitor, led to substantially enhanced replication of the CpG-high and UpA-high mutants (∼90- and 10-fold respectively greater viral loads compared with untreated cells; Figure 13). Furthermore, parallel increases in the infectivity of titred C|C and U|U stocks (Figure 14B) entirely reversed the phenotypic effect of adding CpG and UpA dinucleotides to their genomes and restored RNA to infectivity ratios of these mutants to wild-type levels (Figure 2). Although we initially suspected a role of PKR in mediating the differential cellular response to E7 variants of different dinucleotide composition, several competing observations argue against this hypothesis. Firstly, other inhibitors of PKR expression or function failed to show a comparable enhancement of C|C and U|U replication, including a shRNA and a siRNA directed against PKR and the PKR inhibitor, 2-AP (Figures 12 and 13). Secondly, another kinase inhibitor, roscovitine, also enhanced the replication of C|C and U|U (and strongly suppressed WT virus). This is despite having no identified inhibitory activity against PKR and being largely specific for the cell cycle regulation proteins, CDK1, CDK2, CDK5, CDK7 and CDK9 (46,47). Because C16 has also been shown to also inhibit many of these kinases (27), one might conjecture that the different replication abilities of the E7 mutants may be primarily determined by some aspect of cell cycle regulation.

Replication of at least some enteroviruses is maximally productive at the G1/S stage of the cell cycle (48,49), with very recent evidence that nuclear translocation of enteroviral proteins can prolong the S phase and enhance productive virus replication (50). Roscovitine indeed has the effect of arresting cell division in late G1 through inhibition of cdk2/cyclin E and cdk2/cycle A (46). However, why E7 mutants with increased dinucleotide frequencies are more sensitive to cell cycle stage than WT virus would need to be explained. This model also does not explain why roscovitine and C16 actually inhibited the replication of WT and cu|cu mutants. Their inhibition of cdk2 and cdk5 should arrest cells in the most favourable stage in the cell division cycle for virus replication.

An intriguing alternative possibility is that there exist as yet uncharacterized homologues of stress response proteins that are inhibited by C16 and other kinase inhibitors. Four proteins are currently known to induce stress responses through a common pathway by phosphorylation of eIF2α (51,52). These proteins possess homologous kinases but distinct recognition domains that are activated by different stress factors in the endoplasmic reticulum, including dsRNA (PKR), mis- or unfolded proteins (PERK) and low amino acid concentrations (GCN2). Perhaps there exist one or more additional members of this group that activate the stress response pathway with further recognition motifs (high CpG or UpA RNA) and which are also inhibited by C16 (52). Investigation of cellular binding partners of transfected RNA of different dinucleotide compositions represents a promising future approach to identify what proteins are involved in this recognition process.

### The evolutionary basis for dinucleotide frequency biases in RNA viruses

Although the current study clearly demonstrates the influence of both CpG and UpA frequencies on virus replication in cell culture, it invites as many questions as it answers. The over-riding question is why RNA viruses, with their extraordinary adaptive abilities, have not evolved further to reduce CpG and UpA frequencies to levels lower than they currently are, given the evident replication advantage this provides at least in cell culture (Figures 3, 4, and 6–8). The obvious answer must be that to do so would impair their fitness in their natural hosts through immune response mechanisms not represented in fibroblast cell culture. This may include interactions with specialized antigen-presenting cells and induction of inflammatory and adaptive immune responses that primarily drive the systemic response to and eventual outcomes of virus infections. Alternatively, enhanced replication may somehow impair transmission kinetics in the host population. We have established equivalent panels of high- and low CpG/UpA mutants of Theiler’s murine encephalomyelitis virus and H1N1 influenza A virus for infection of mice to investigate the effect of dinucleotide frequency changes on *in vivo* fitness. The key question to explore is the behaviour of mutants with lowered dinucleotide frequencies, whether they achieve greater or lesser degrees of replication *in vivo* and separately whether they are attenuated or show enhanced pathogenicity. Although not formally investigated to date, if we take the previously described PV-Max mutant as an example of a virus with suppressed CpG and UpA frequencies (34), the observed threefold greater viral titres in brain of peripherally inoculated mice seems to imply that their suppression confers an *in vivo* fitness advantage.

Further *in vivo* studies are clearly required to place dinucleotide frequencies into an appropriate evolutionary and immunological framework. There is an additional need to further characterize effects of other nucleotide and dinucleotide compositional abnormalities of RNA virus genomes on cellular response. This applies in the current study to the unexpected enhanced replication of the U|W E7 mutant (Figure 3) that contrasted with the otherwise primarily dose-dependent relationship between dinucleotide composition and replication ability in the current study (Figures 3 and 4) and in previous mutational analyses of poliovirus (17). Recent investigations of the effect of biased mononucleotide compositions of human immunodeficiency virus type 1 on cellular responses provide evidence for further complexities in the interaction between the viral genomic RNA sequences and host defences (53,54).

A wide range of other issues remain to be resolved. While changing UpA frequencies mirrored at least in part phenotypes induced by altering CpG frequencies, it remains unclear whether they share recognition mechanisms and/or susceptibilities to cellular responses. For example, UpA (along with UpU) but not CpG is a preferred target for IFN-induced RNAseL (55,56) and plays a determining role in mRNA turnover rates (57). Replication of high UpA mutants was enhanced less by C16 (Figures 13 and 14) providing some evidence that cellular recognition mechanisms underlying the replication phenotypes of U|U and C|C mutants were distinct. Similarly difficult to account for is the observation that CpG (and UpA) frequencies are markedly suppressed in plant viruses even though viral defence mechanisms in plants are based on entirely different recognition, signalling and effector pathways from those in mammals. Uncovering the nature of selection against these dinucleotides in plants will be highly informative in further understanding evolutionary processes that govern dinucleotide compositional constraints in a wider eukaryotic paradigm.

## SUPPLEMENTARY DATA

Supplementary Data are available at NAR Online.

## FUNDING

Wellcome Trust [WT087628MA]; Programme Grant from the MRC [G0401584]. Funding for open access charge: University of Edinburgh.

*Conflict of interest statement*. None declared.

## Supplementary Material

Supplementary Data
