# Supplementary material for: The influence of CpG and UpA dinucleotide frequencies on RNA virus replication and characterization of the innate cellular pathways underlying virus attenuation and enhanced replication
Source: Nucleic Acids Res. 2014 Jan 25;42(7):4527–45. doi: 10.1093/nar/gku075 (PMC3985648; doi:10.1093/nar/gku075)
Supplement: Supplementary Data [file supp_42_7_4527__index.html]

The influence of CpG and UpA dinucleotide frequencies on RNA virus replication and characterization of the innate cellular pathways underlying virus attenuation and enhanced replication — Supplementary Data 

# The influence of CpG and UpA dinucleotide frequencies on RNA virus replication and characterization of the innate cellular pathways underlying virus attenuation and enhanced replication

## Supplementary Data

files

**Files in this Data Supplement:**

- Supplementary Data - pdf file
