# Supplementary material for: The influence of CpG and UpA dinucleotide frequencies on RNA virus replication and characterization of the innate cellular pathways underlying virus attenuation and enhanced replication
Source: Nucleic Acids Res. 2014 Jan 25;42(7):4527–45. doi: 10.1093/nar/gku075 (PMC3985648; doi:10.1093/nar/gku075)
Supplement: Supplementary Data [file supp_gku075_nar-03011-v-2013-File002.pdf]

Supplementary Data

Table 1

Primers used in qRT-PCR

Gene	Sense	Antisense
E7 5' UTR	TCCGGCCCCTGAATGCGGCTAA	CACCCAAAGTAGTCGGTTCCGC
GAPDH	GAAATCCCATCACCATCTTCCAGG	GAGCCCCAGCCTTCTCCATG
ISG15	GGCGGGCAACGAATTCCAGGTGT	CTCCCCGCAGGCGCAGATTCA
ISG56	CTTGAGCCTCCTTGGGTTCG	GCTGATATCTGGGTGCCTAAGG
RIG-I	TCCAGATGCCAGACAAAGATGAAG	CCTGCTGCTCGGACATTGC
MDA-5	CGCTATCTCATCTCGTGCTTCAG	TCCAACCAAGGTGCCAGACTC
PKR	CCCAGATTTGACCTTCCTGA	ACTTGGCCAAATCCACCTG

Nested primers used in sequencing individual viral genomes

Region	Virus	Primer type	Nucleotide position	Sequence
1	All	Outer, sense	1809	CCCAATTTGATGTAACACCACACATGG
1	All	Inner, sense	1835	GATATTCCAGGCGAAGTACACAACC
1	W|W	Outer, antisense	2343	CAAAGCACTACACACTTATTTGGAG
1	C|C	Outer, antisense	2382	ATTCGAACGGAGAAATCGTTAC
1	U|U	Outer, antisense	2388	TCCCTTAGCATACGTACTGAGAAAT
1	W|W	Inner, antisense	2313	GCACCACTATTCCTGTTTGGT
1	C|C	Inner, antisense	2348	AACAAAGCACGACGCACTTATT
1	U|U	Inner, antisense	2363	CATTACAAGCTGATGCAAAACATAG
Un-modified	All	Outer, sense	3210	TGAGCCCGTACATCAAATCA
Un-modified	All	Inner, sense	3241	TTTTAACCCCACGAACCTGA
Un-modified	All	Outer, antisense	3785	TTGCCGAGTTGTTCGACATA
Un-modified	All	Inner, antisense	3723	CAAGTCACGGATGTCTGCAA

Nested primers used in amplification of E7 Region 1 or Region 2 for competition assays

Region	Primer	Sense	Antisense
1	Outer	CCCAATTTGATGTAACACCACACATGG	CCCATACTCGGATGTGCTTGGG
1	Inner	GATATTCCAGGCGAAGTACACAACC	CACTCGGATTGTGCTTGACATCTG
2	Outer	CAAGGAGCATACACAGGAATACC	GAATGTCTGCCTCATCGCCAACT
2	Inner	GGTACCTACTCTTAGGCAAGCA	AAGCTGGACGCTTCAATGAGCCT

Table S2

Enzymes used in selective digests for competition assays

Virus 1	Virus 2	Region amplified	Enzyme	Restriction site
**Individual competition experiments**				
W|W	P|P	2	HindIII	In R1/R2 Permuted
W|W	C|C	1	BamHI	In R1/R2 CpG-high
W|W	U|U	2	ScaI	In R1/R2 UpA-high
W|W	c|c	2	SphI	In R1/R2 CpG-low
W|W	u|u	2	EcoRV	In WT
**Pairwise competition experiments**				
W|W	P|P	2	HindIII	In R1/R2 Permuted
W|W	cu|W	1	EcoRV	In WT
W|W	W|cu	2	EcoRV	In WT
W|W	c|c	2	EcoRV	In R1/R2 CpG-low
W|W	u|u	2	EcoRV	In WT
W|W	cu|cu	2	SphI	In WT
P|P	cu|W	1	SphI	In R1/R2 Permuted
P|P	W|cu	2	HindIII	In R1/R2 Permuted
P|P	c|c	2	HindIII	In R1/R2 Permuted
P|P	u|u	2	HindIII	In R1/R2 Permuted
P|P	cu|cu	2	HindIII	In R1/R2 Permuted
cu|W	W|cu	1	EcoRV	R2 CpG/UpA-low
cu|W	c|c	2	SphI	In R1/R2 CpG-low
cu|W	u|u	2	EcoRV	In R1 CpG/UpA-low
cu|W	cu|cu	2	EcoRV	In R1 CpG/UpA-low
W|cu	c|c	2	EcoRV	In R1/R2 CpG-low
W|cu	u|u	2	SphI	In R2 CpG/UpA-low
W|cu	cu|cu	1	EcoRV	In R2 CpG/UpA-low
c|c	u|u	2	EcoRV	In R1/R2 CpG-low
c|c	cu|cu	2	EcoRV	In R1/R2 CpG-low
u|u	cu|cu	2	SphI	In R1/R2 CpG/UpA-low

Table S3

Nucleotide substitutions observed after sequencing individual passage 1 virus genomes.

Virus	Region	kb sequenced	Changes observed	Nucleotide position	Type	Changes in CpG/UpA
E7 WT	1	7	None			
E7 WT	Un-modified	8.5	None			
R1/R2 CpG high	1	7.5	C→A	1963	Synonymous	None
R1/R2 CpG high	Un-modified	7.5	A→C<br>C→U	3665<br>3247	Synonymous<br>Synonymous	CpG created<br>None
R1/R2 UpA high	1	8	A→G<br>U→C	2275<br>2193	Synonymous<br>Non-synonymous: M→T	None<br>CpG created
R1/R2 UpA high	Un-modified	7.5	None			

Figure S1

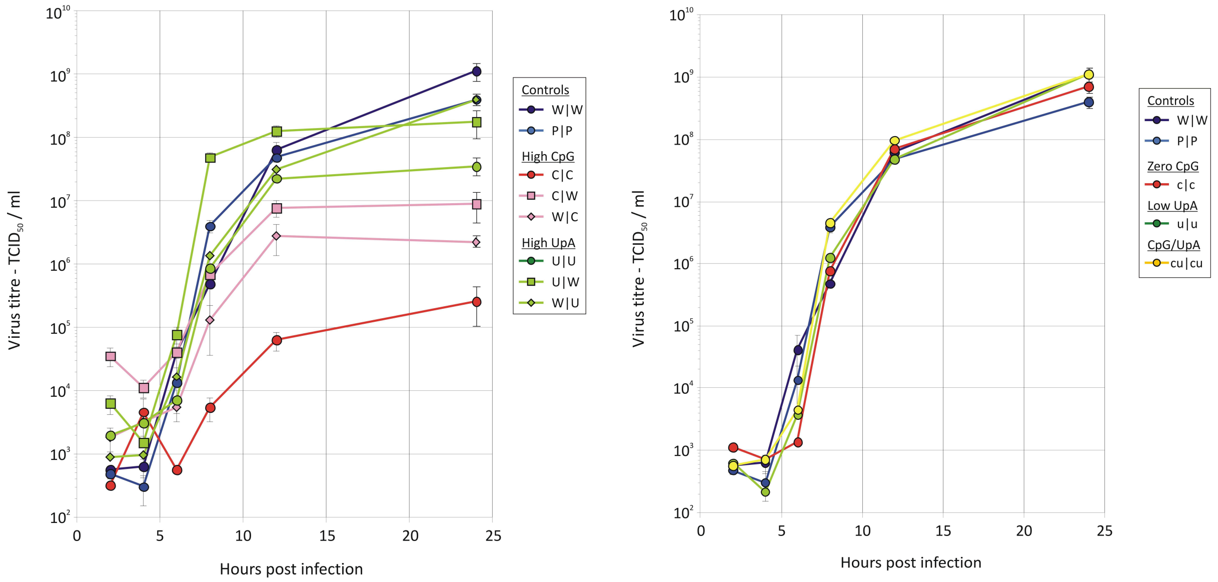

Single-step replication assay for E7 and mutant viruses. RD cells were infected with E7 WT, Permuted, CpG/UpA-high mutants (**A)** or CpG/UpA-low mutants (**B**) at an MOI of 10. Infectious titre of supernatants was measured at different time points by $TCID_{50}$ titrations Data points are the mean of three biological replicates (SEMs shown by error bars).

Figure S2

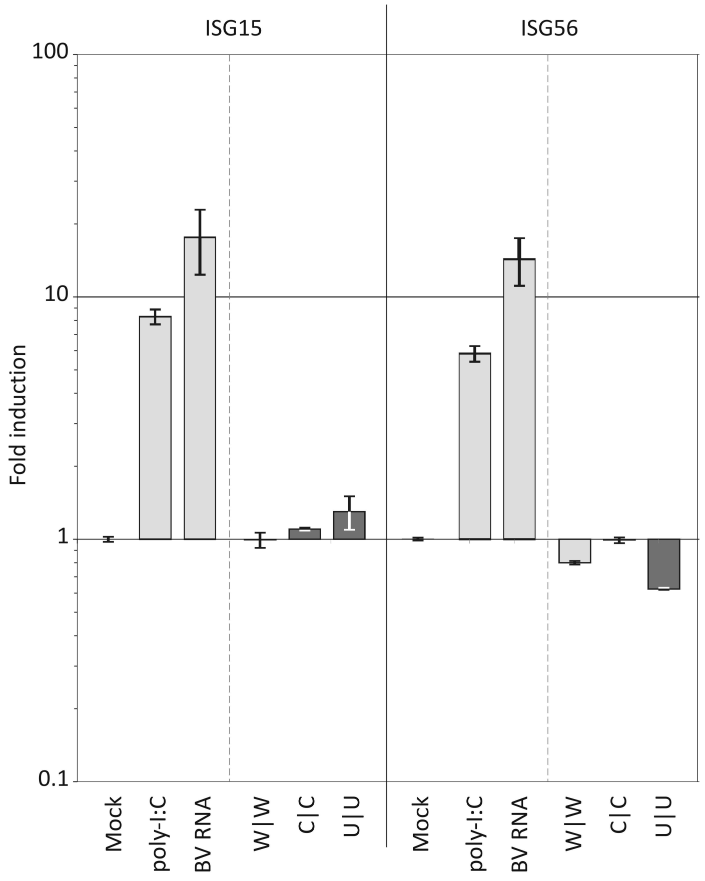

Expression of ISG15 and ISG56 following infection with WT or mutant virus. Gene expression was analysed by qRT-PCR 8 hours following infection with E7, C|C or U|U mutants at an MOI of 10. Poly I:C was transfected as a positive control. Expression is normalised to GAPDH and is shown relative to the E7 WT value. Results are the mean and standard error of two biological replicates.

Figure S3

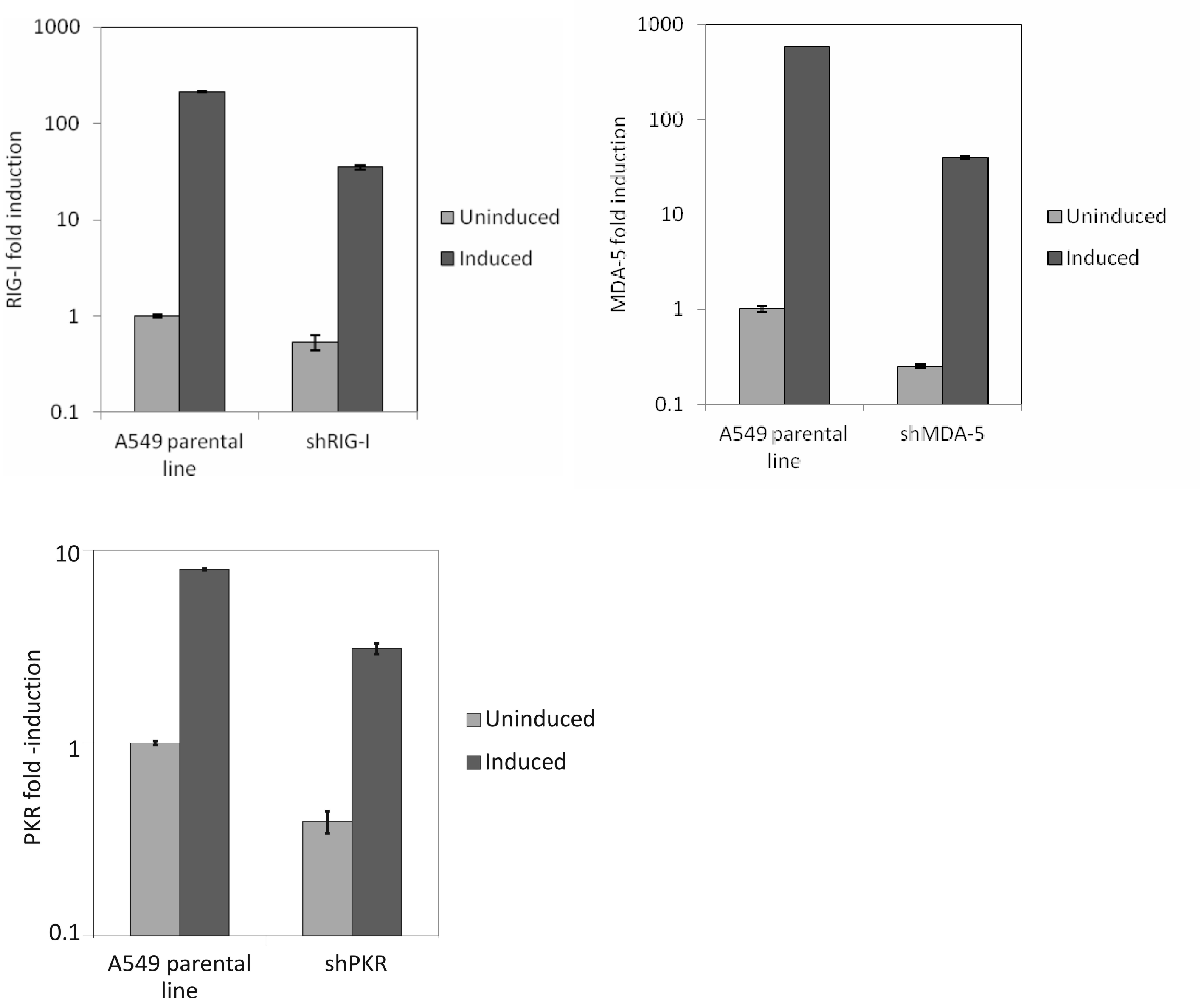

Expression of RIG-I, MDA-5 and PKR in induced and uninduced shRNA lines. Cell lines expressing RIG-I, MDA-5 or PKR shRNAs, as well as the parental A549 cell line, were transfected with Poly I:C for 8 hours and the relative expression levels of the targeted gene quantified using qRT-PCR. Expression was normalised against GAPDH and is shown relative to the uninduced parental cell line value. Results are the mean and standard error of two technical replicates.

Figure S4

A)

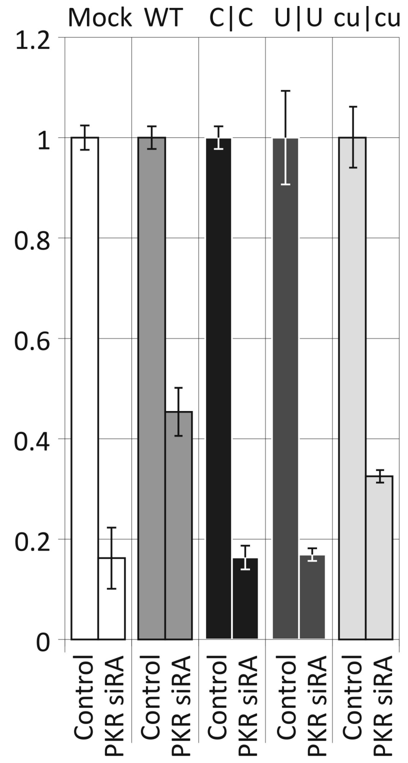

B)

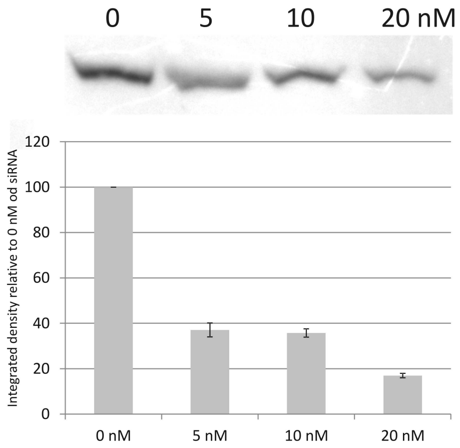

A) Reduction in PKR mRNA levels by 44 hour pre-treatment with PKR siRNA; reductions shown relative to pre-treatment with a validated non-targeted control siRNA. Reductions in PRK mRNA expression were observed irrespective of whether cells were infected with E7 or not.

B) Detection of PKR by Western blot in cell treated with PKR siRNA. Cells were transfected with siRNAs to PKR and incubated for 48 hours. Equal amounts of protein in cell lysates were blotted and detected using a PKR specific mAB followed by anti-rabbit HRP conjugated antibodies and ECL detection. Band densities were measured for two independent experiments. A dose-dependent reduction of protein expression by up to 83% (20nM) was observed.
